# Supplementary material for: Mutations affecting the N-terminal domains of SHANK3 point to different pathomechanisms in neurodevelopmental disorders
Source: Sci Rep. 2022 Jan 18;12:902. doi: 10.1038/s41598-021-04723-5 (PMC8766471; doi:10.1038/s41598-021-04723-5)

Mutations affecting the N-terminal domains of SHANK3 point to different pathomechanisms in neurodevelopmental disorders.

Daniel Woike, Emily Wang, Debora Tibbe, Fatemeh Hassani Nia, Antonio Virgilio Failla, Maria Kibæk, Tinett Martesen Overgård, Martin J. Larsen, Christina R. Fagerberg, Igor Barsukov, Hans-Jürgen Kreienkamp

Supplemental data.

Patient data.

Individual III:1. The index patient is a boy aged 12 years who born as the first child of non-consanguineous parents. He has a younger sister. He was born at gestational age 36+0 weeks with a birth weight of 2566 g, length 50 cm, head circumference 32 cm, and Apgar score 10/1, 10/5. He had from early on a poor sleep with several nightly awakenings. He walked at age 1 year and had recurrent middle ear infections. He was referred to the pediatric department at age 2½ years due to overweight, poor balance, limited language, difficulties with concentration, and an aggressive outward reacting behavior. He started in a special group in nursery, and he attends a school for children with special needs. At age 6½ years he was seen with premature adrenarche. A slight hypermetropia was noted. He lives in an institution and has done that in periods since age 6 years. A WPSSI-R test at age 4 years showed a total IQ of 92. A WISC-IV-test at age 11 years showed a total IQ of 73 with an uneven profile extending from 66 for processing speed to 97 for reasoning. The low threshold for frustration was suspected to influence the test results. He does not fulfill the criteria for ADHD, but has an outward reacting behavior with a tendency of being involved in fights, demanding avoidance, irritability, and uncontrollable behavior. He is evaluated to fulfill the criteria for Oppositional Defiant Disorder. He needs structure and a predictable day. Current height is 171.5 cm (+2.5 SD), weight 90.5 kg (+3.2 SD), resulting in a Body mass index (BMI) of 30.8. Head circumference at age 10 y 4 m was 55 cm (approximately +1.5 SD).

Investigation for Fragile X and methylation specific MLPA for Prader Willi Syndrome showed normal result. Chromosomal microarray at age 3 years showed a maternally inherited duplication in Xp11.14 of 140 kb with a breakpoint in *TSPAN7*, the variant was later evaluated to be a rare normal variant without phenotypic consequence. Duo-exome analysis revealed the maternally inherited missense variant *SHANK3* (NM_033517.1):c.808C>A, p.(Leu270Met).

Individual II:2 is the mother of individual III:1, her current age is 32 years. She had severe overweight since childhood. She was a problematic child in school with outward reacting behavior. There were some learning difficulties, but she has taken elementary school exam. Cognitive testing was not done. She had cholecystitis with cholecystectomia at age 21 years. She is diagnosed with ADHD, for which she receives medical treatment with good effect, and also with an emotional instable personality borderline disorder (SCIDII-interview). At age 31 years she had surgery for bilateral carpal tunnel syndrome. She is severely overweight, with height 176 cm, and weight 137 kg, resulting in a BMI of 44.

Individual II:4 is the brother of individual II:2, his current age is 30 years. He has since early childhood had an outward reacting behavior, and had a problematic time in school. From 5th grade, he was absent from school most of the time. He had difficulties in concentration, and learning disabilities especially in mathematics, while he has performed well in language subjects after medication (as an adult). He has had periodical abuse of alcohol, cannabis and narcotics since teenage years. He was diagnosed with ADHD at approximately 20 years, medical treatment had good effect. He has been diagnosed with Wolf-Parkinson-White. He has been sentenced to prison twice due to theft and weapon possession respectively. He has been overweight in periods. His present height is 185 cm and weight 114 kg, resulting in a BMI of 33.

Individual I:2 is the mother of individuals II:2 and II:4. Her current age is 55 years. She has not been seen at the department, but gave the following information by telephone: She was hot-tempered when she was younger, but states that she did well at school. She is not overweight. The childhood home was marked by drugs, alcohol and beating. She has four halfsiblings (all with the same mother as herself, one half-brother and two half-sisters had one same father, one half-sister had another father), whom she think all had difficulties in school. The half-brother had a bad temper, and one half-sister had mood swings. She did not wish to involve her side of the family in the genetic evaluation.

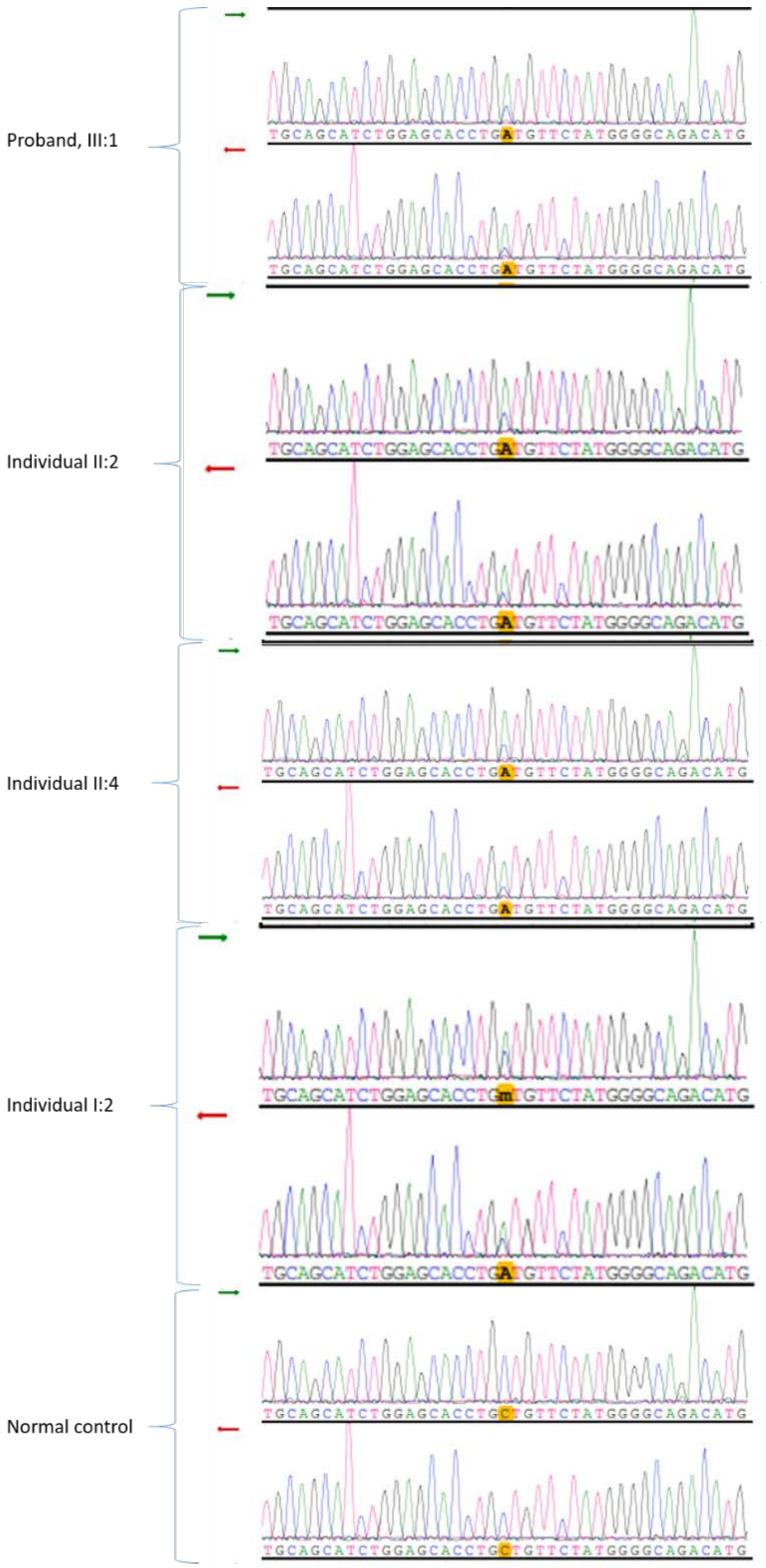

Supplementary Fig. S1. Electropherograms from Sanger sequencing showing the heterozygous variant SHANK3:c.808C>A,p.Leu270Met.

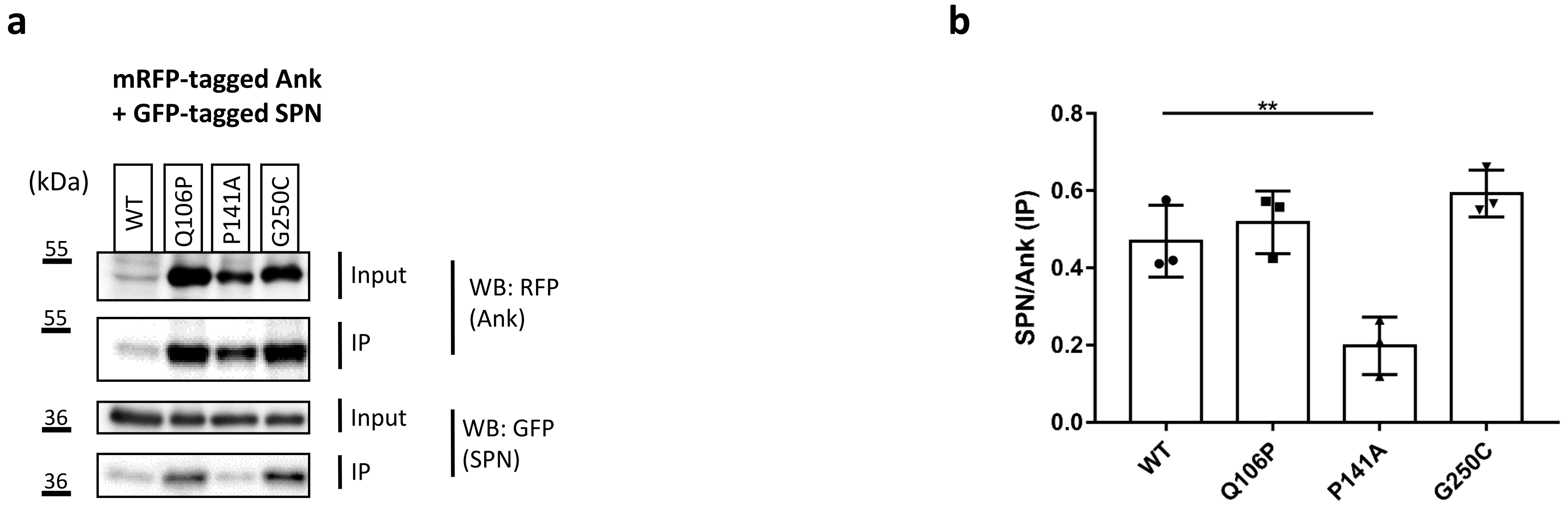

Supplementary Fig. S2 a. RFP-tagged WT and mutant variants of the Shank3 Ank repeats (shorter Ank construct including residues 95 - 339) were coexpressed in 293T cells with the GFP-tagged SPN domain of Shank3. After cell lysis, RFP-tagged proteins were immunoprecipitated using the mRFP-trap matrix. Input and precipitate samples were analysed by Western blotting using mRFP- (upper panels) and GFP-specific antibodies (lower panels). **b**. Quantitative analysis. Signal intensities in IP samples for GFP-SPN were divided by IP signals for mRFP-Shank3 variants. **, significantly different from WT, p<0.01; ANOVA, followed by Dunnett's multiple comparisons test.

Shank3	VARLLDKGLD**P**NFHDPDSGEC
Shank2	ITKMLDRGLDPNFHDPETGET
Shank1	VARLLDKGLDPNYHDSDSGET
Drosophila	IAKMCAKGLDPNFHCSESGDT

Supplementary Figure S3. Sequence alignment of different Shank proteins around the position of proline 141 in Shank3 (bold print), showing high sequence conservation.

Supplementary Information

Original blot images

Mutations affecting the N-terminal domains of SHANK3 point to different pathomechanisms in neurodevelopmental disorders.

Daniel Woike, Emily Wang, Debora Tibbe, Fatemeh Hassani Nia, Antonio Virgilio Failla, Maria Kibæk, Tinett Martesen Overgård, Martin J. Larsen, Christina R. Fagerberg, Igor Barsukov, Hans-Jürgen Kreienkamp

Fig. 2 a.

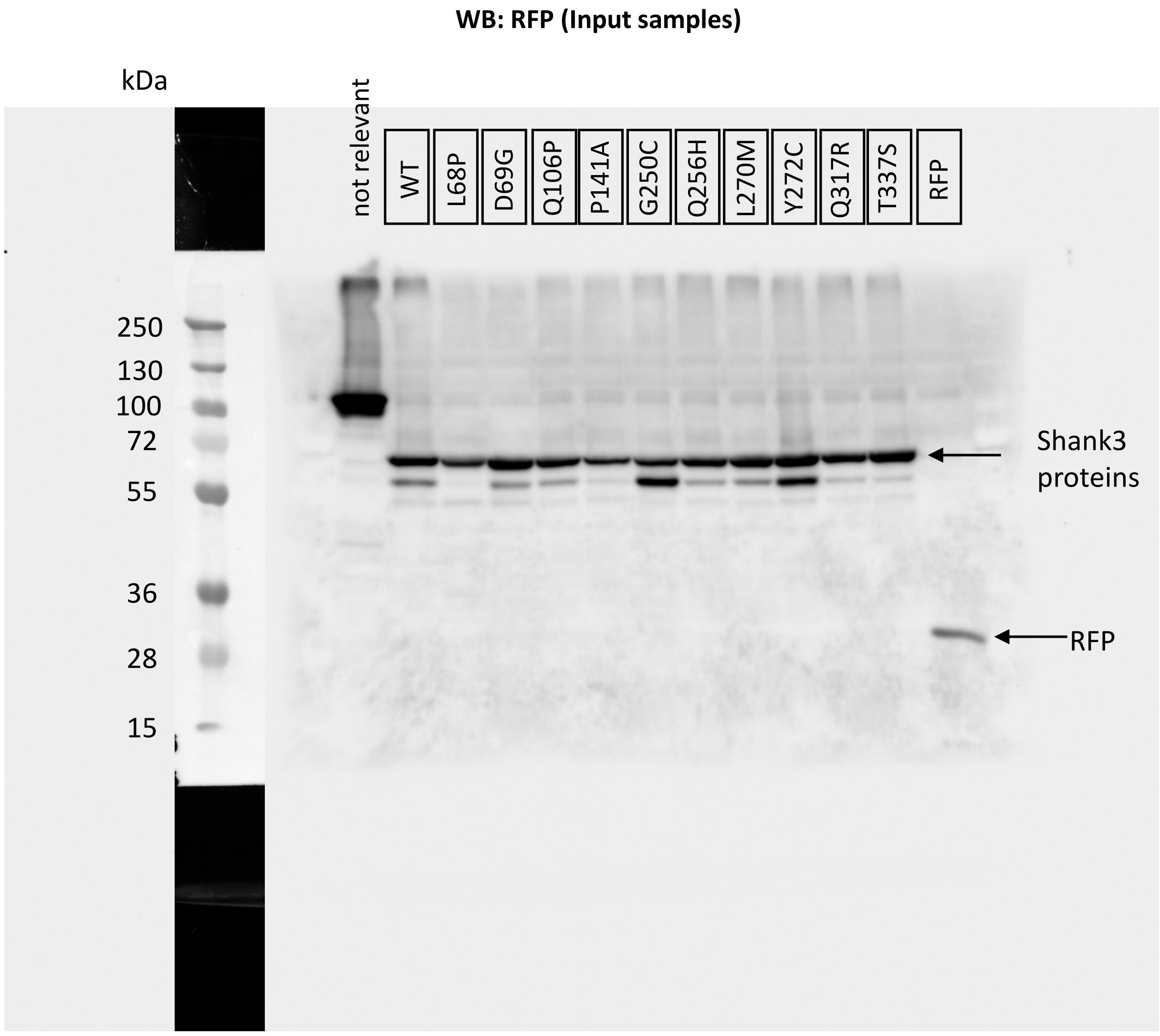
WB: RFP (Input samples)
kDa
not relevant
WT
L68P
D69G
Q106P
P141A
G250C
Q256H
L270M
Y272C
Q317R
T337S
RFP
250
130
100
72
55
36
28
15
Shank3
proteins
RFP

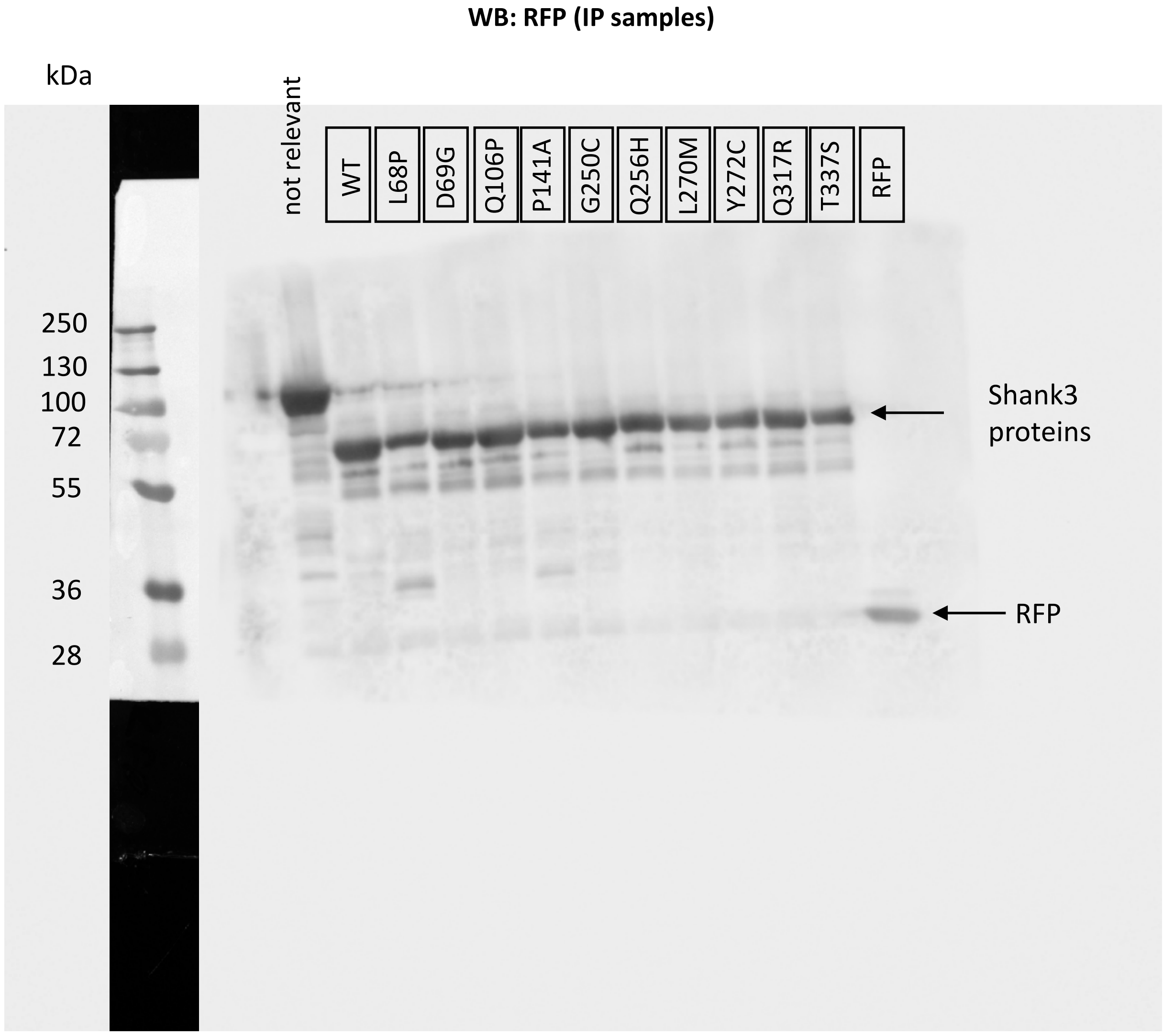
WB: RFP (IP samples)
kDa
not relevant
WT
L68P
D69G
Q106P
P141A
G250C
Q256H
L270M
Y272C
Q317R
T337S
RFP
250
130
100
72
55
36
28
Shank3
proteins
RFP

Fig. 2 a.

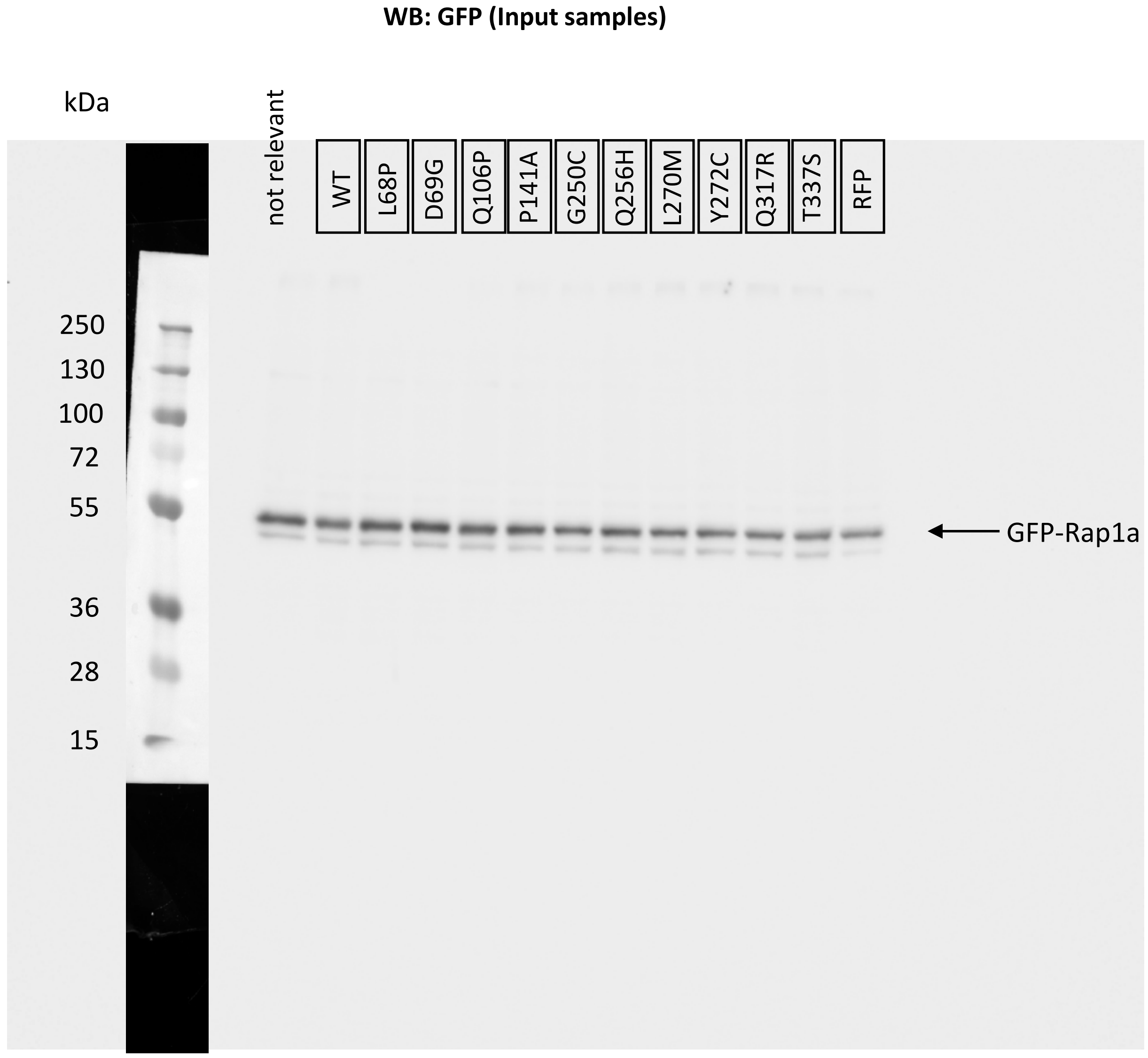
WB: GFP (Input samples)
kDa
not relevant
WT
L68P
D69G
Q106P
P141A
G250C
Q256H
L270M
Y272C
Q317R
T337S
RFP
250
130
100
72
55
36
28
15
GFP-Rap1a

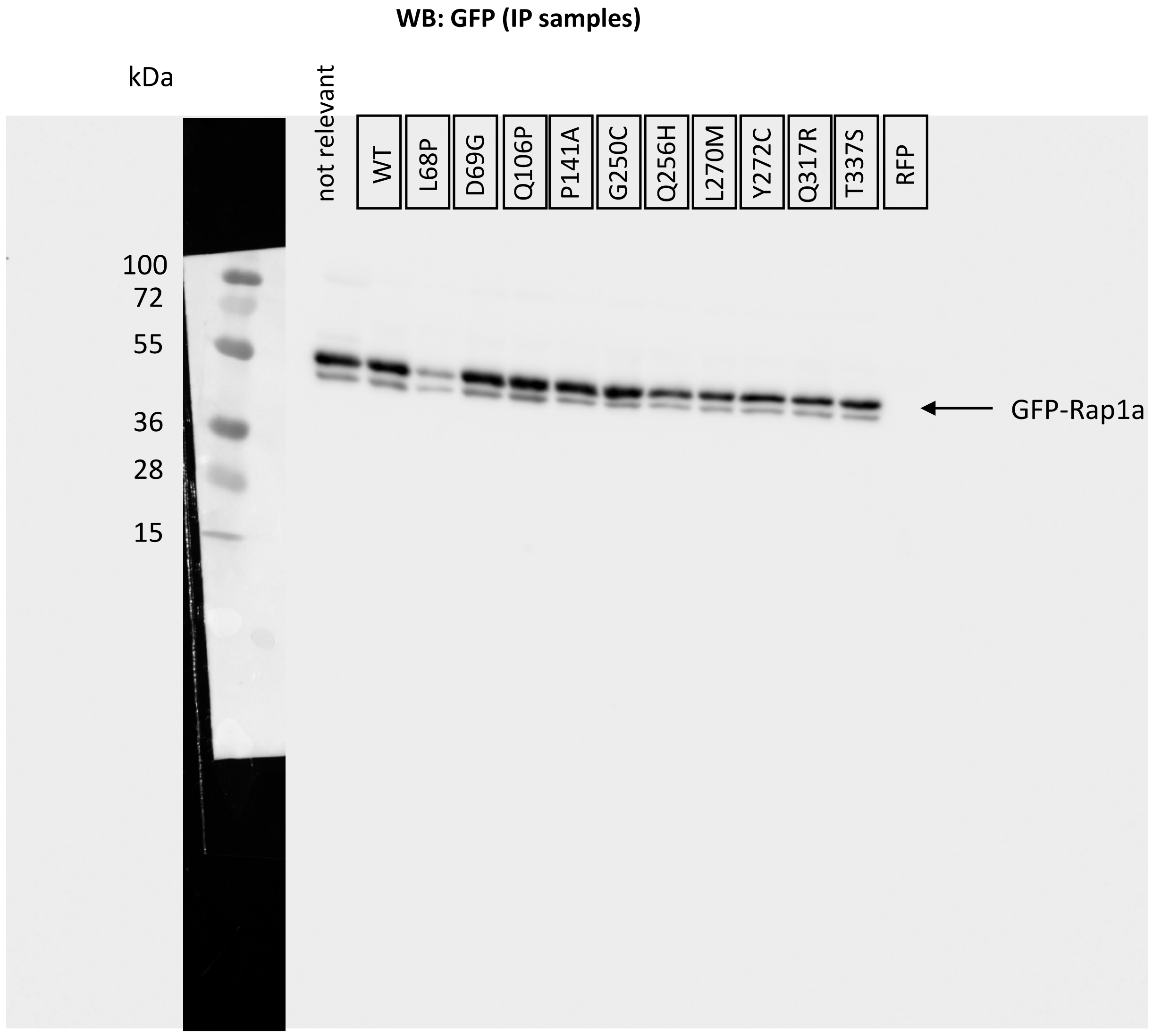
WB: GFP (IP samples)
kDa
not relevant
WT
L68P
D69G
Q106P
P141A
G250C
Q256H
L270M
Y272C
Q317R
T337S
RFP
100
72
55
36
28
15
GFP-Rap1a

Fig. 2 b.

WB: RFP (Input samples)

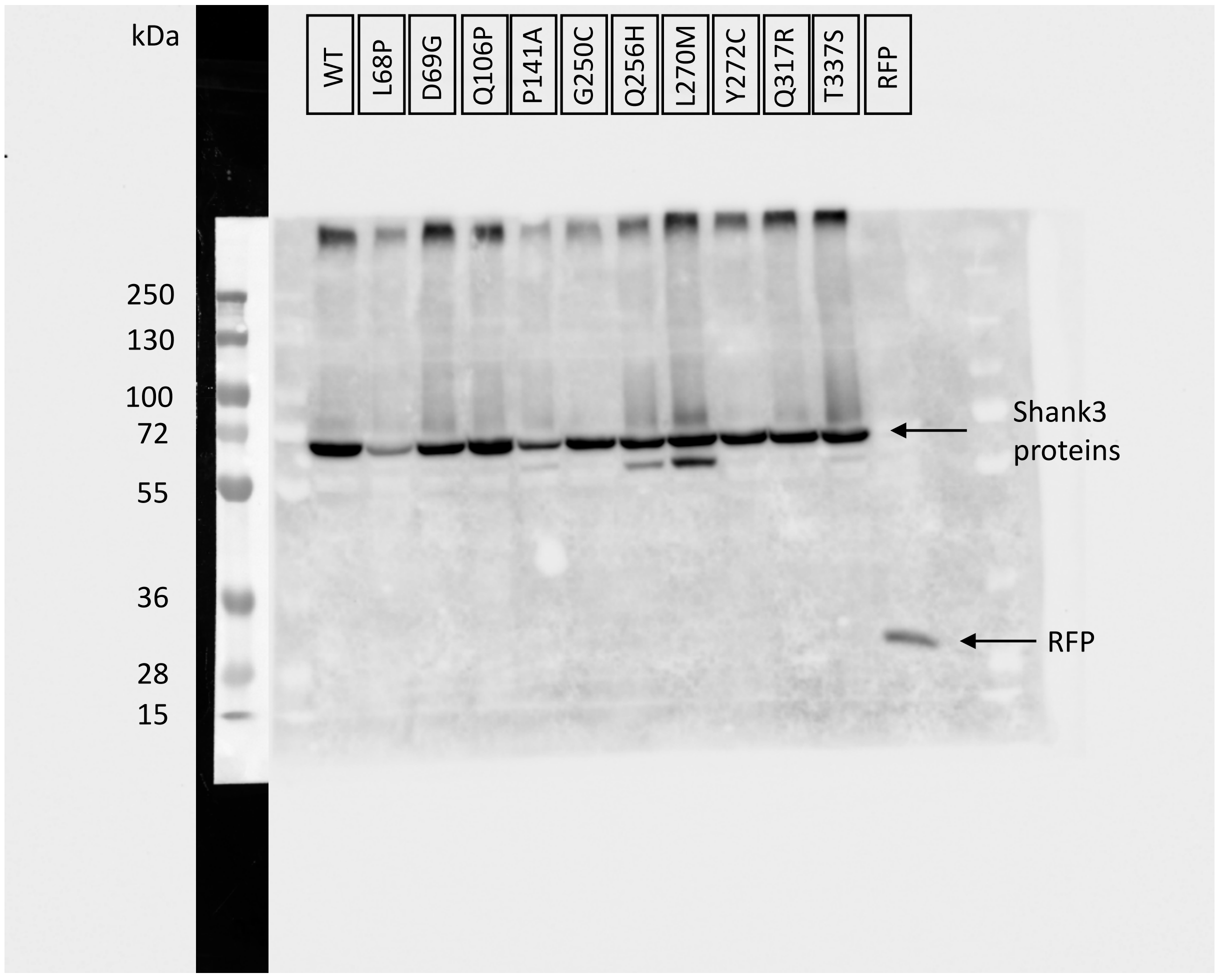

WB: RFP (IP samples)

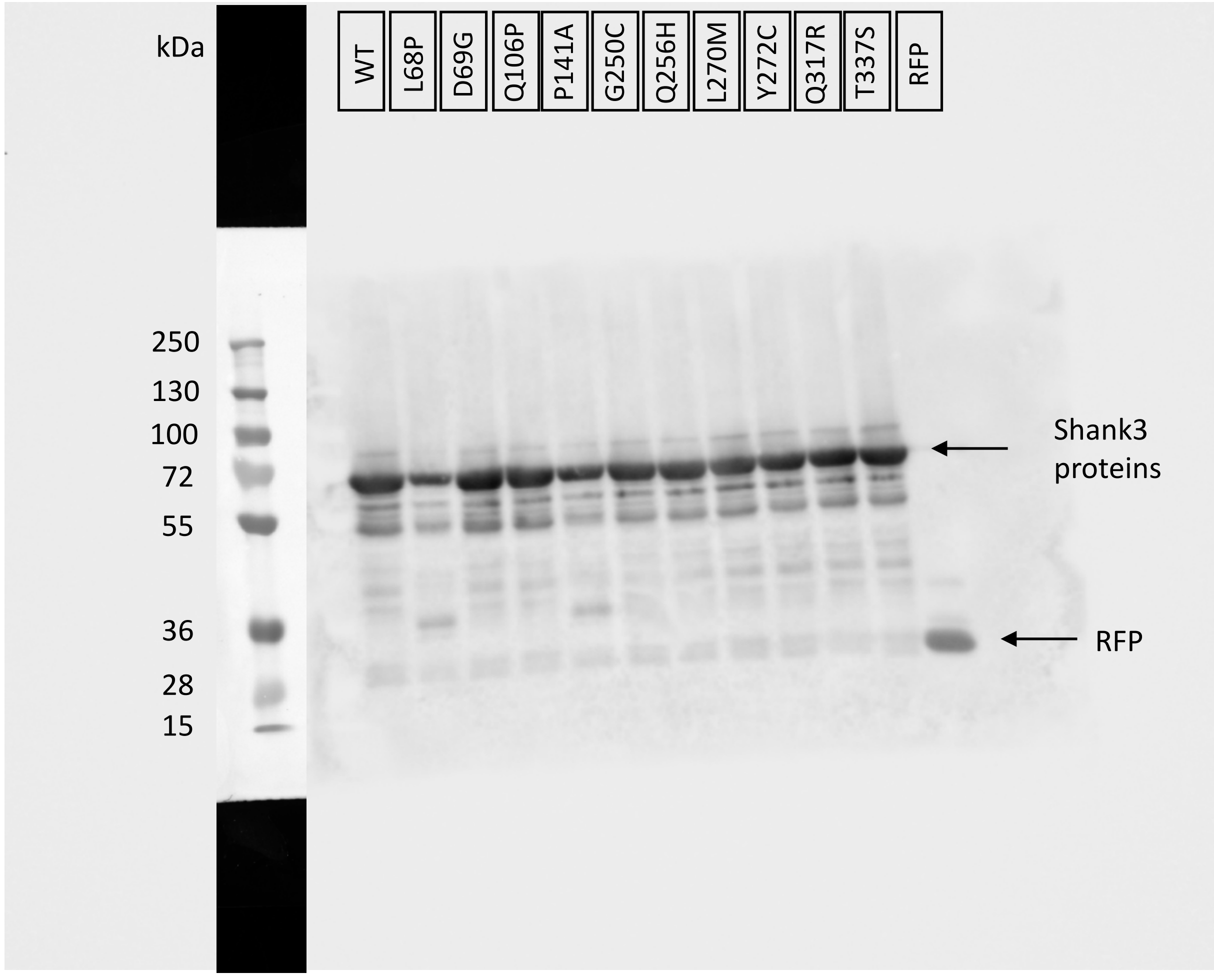

Fig. 2 b.

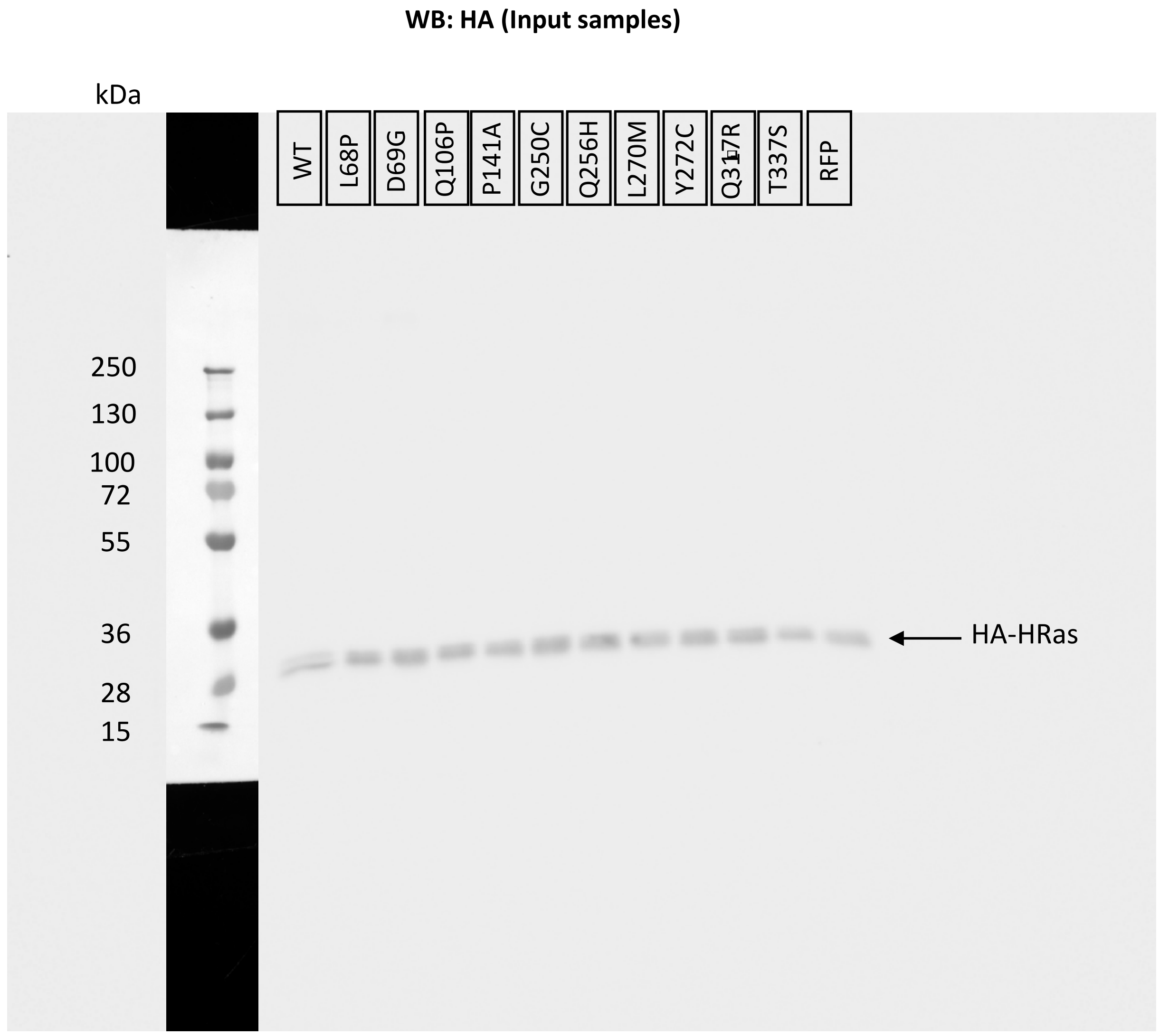

WB: HA (Input samples)
kDa
WT
L68P
D69G
Q106P
P141A
G250C
Q256H
L270M
Y272C
Q317R
T337S
RFP
250
130
100
72
55
36
28
15
HA-HRas

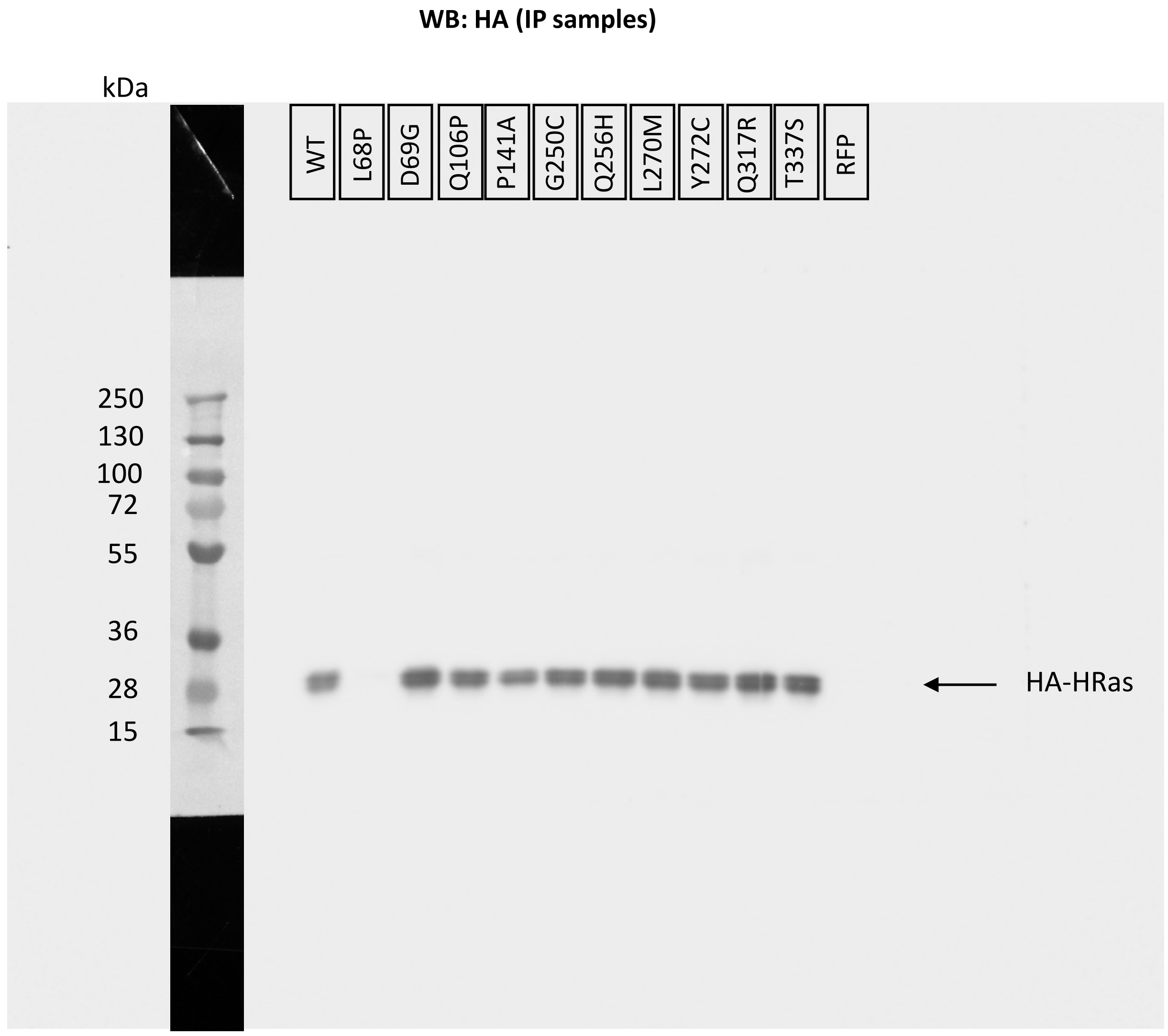

WB: HA (IP samples)
kDa
WT
L68P
D69G
Q106P
P141A
G250C
Q256H
L270M
Y272C
Q317R
T337S
RFP
250
130
100
72
55
36
28
15
HA-HRas

Fig. 3 a.

WB: RFP (Input samples)

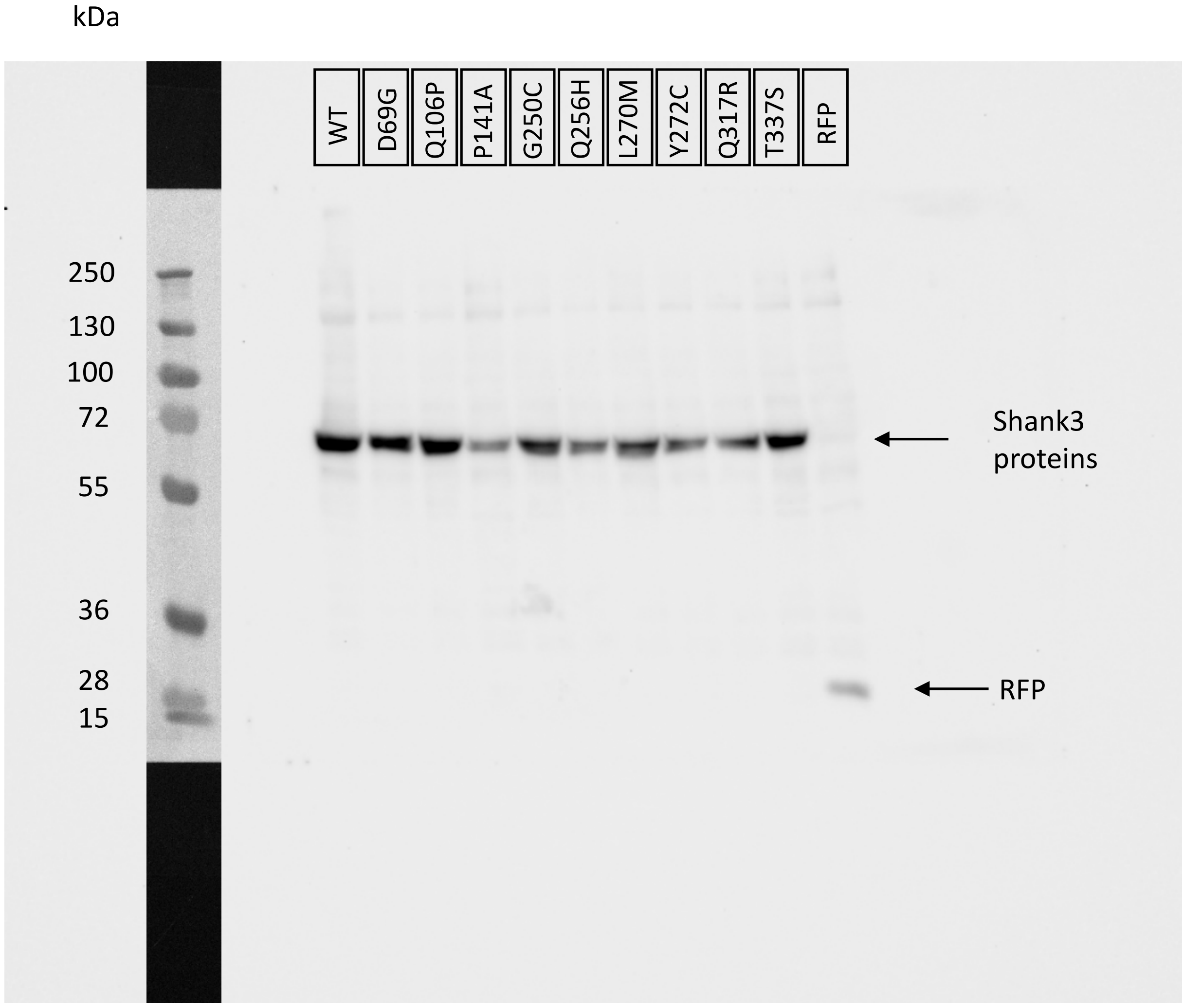

WB: RFP (IP samples)

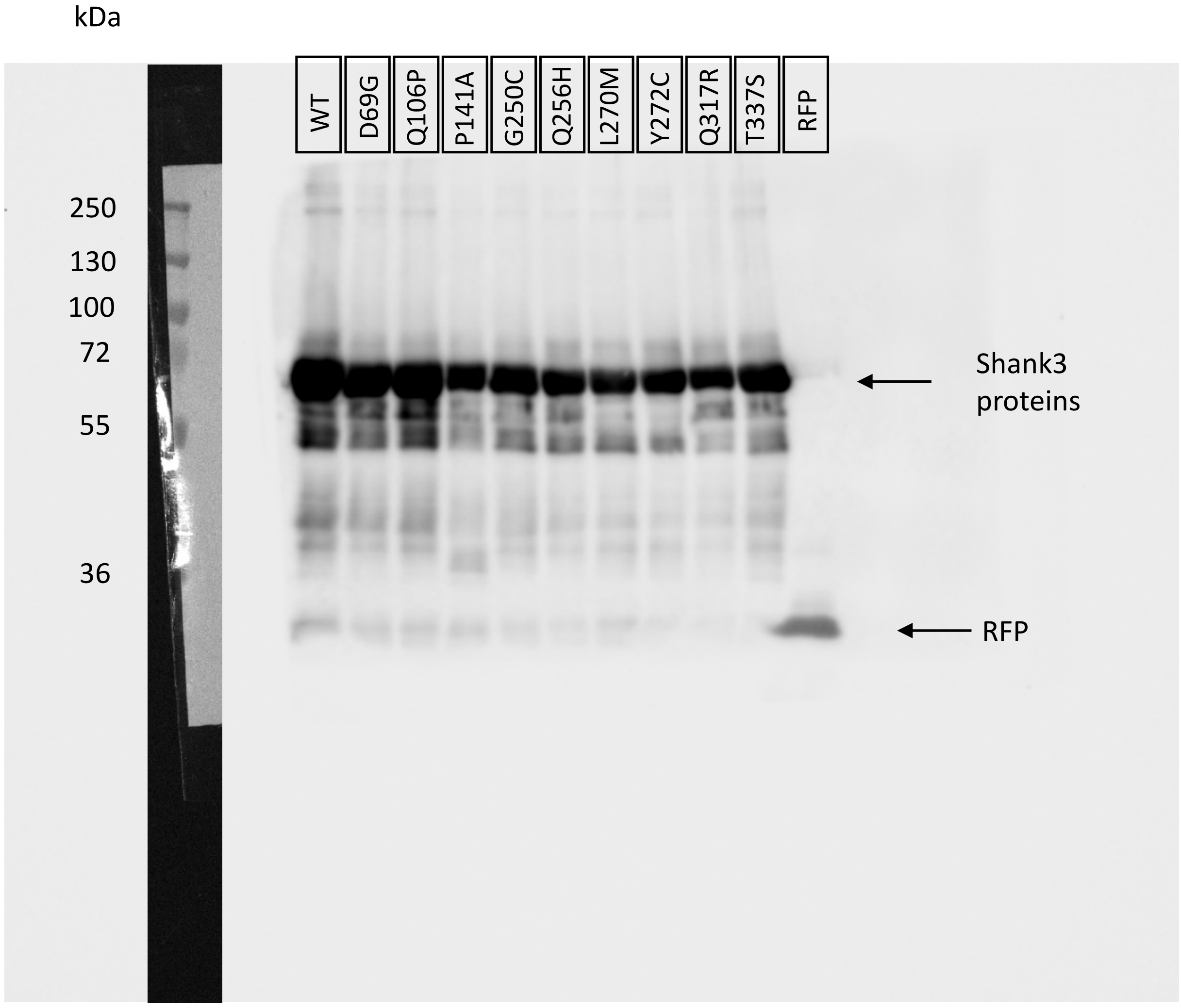

Fig. 3 a.

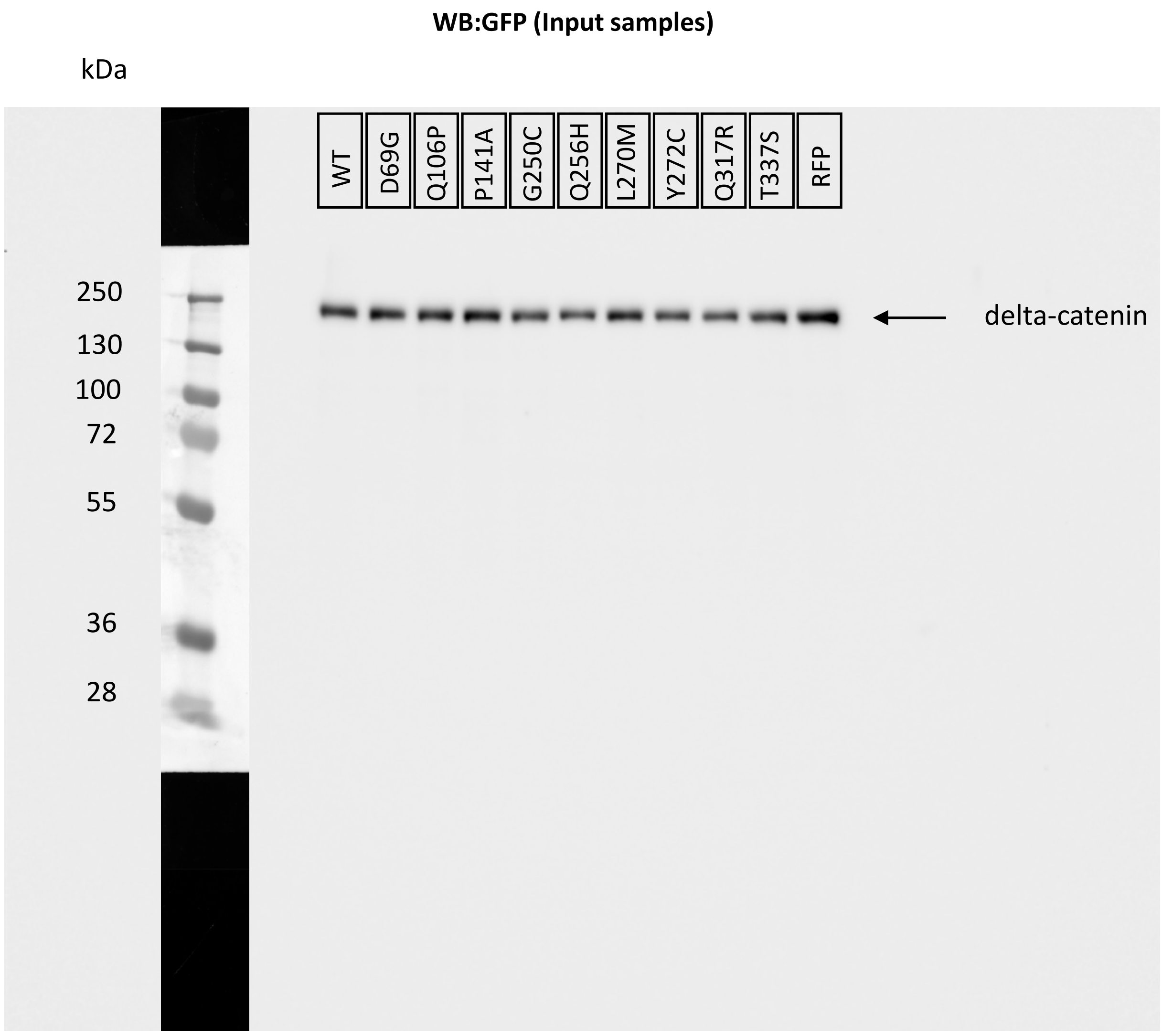
WB:GFP (Input samples)
kDa
250
130
100
72
55
36
28
WT
D69G
Q106P
P141A
G250C
Q256H
L270M
Y272C
Q317R
T337S
RFP
delta-catenin

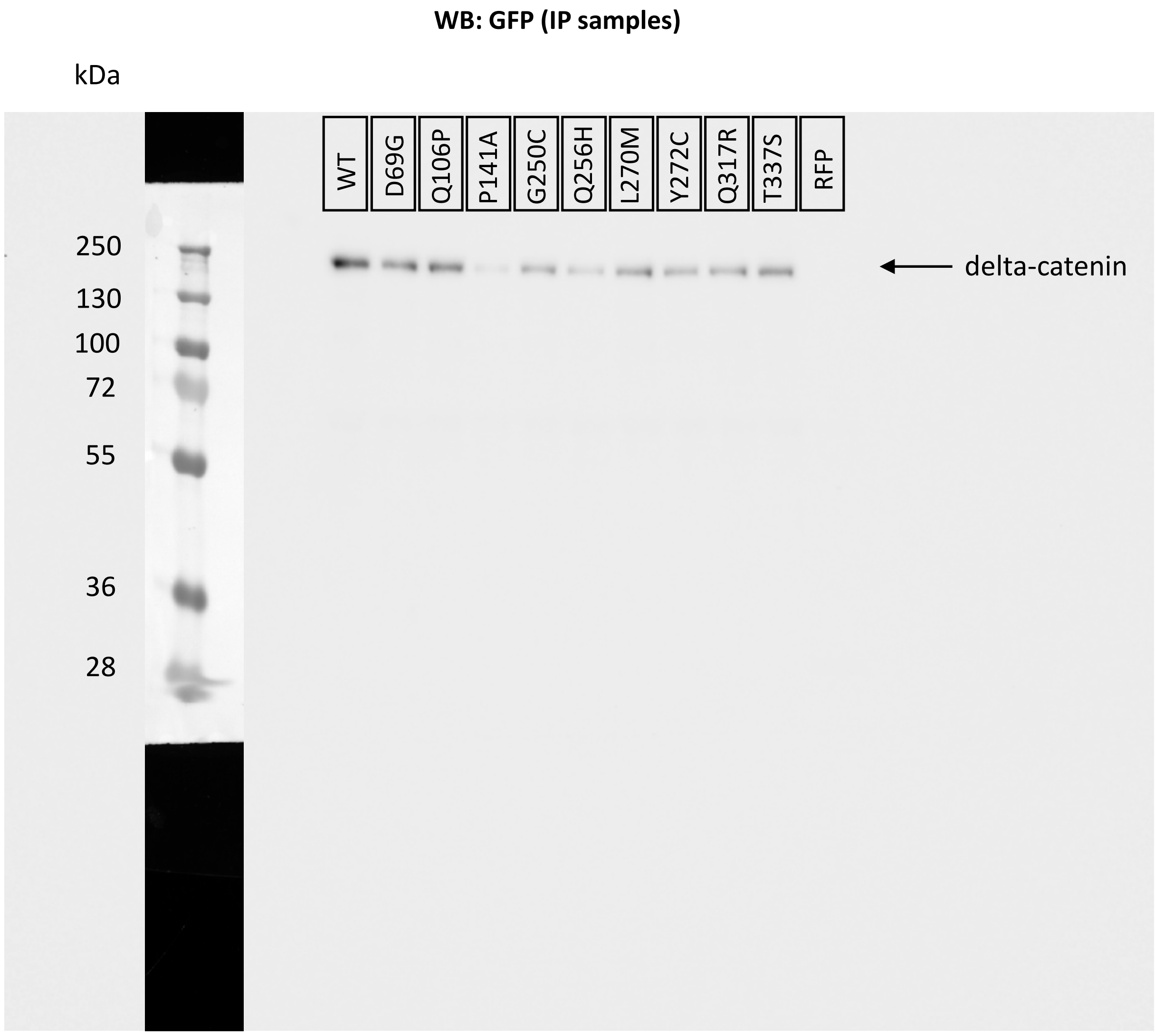
WB: GFP (IP samples)
kDa
250
130
100
72
55
36
28
WT
D69G
Q106P
P141A
G250C
Q256H
L270M
Y272C
Q317R
T337S
RFP
delta-catenin

Fig. 4 a.

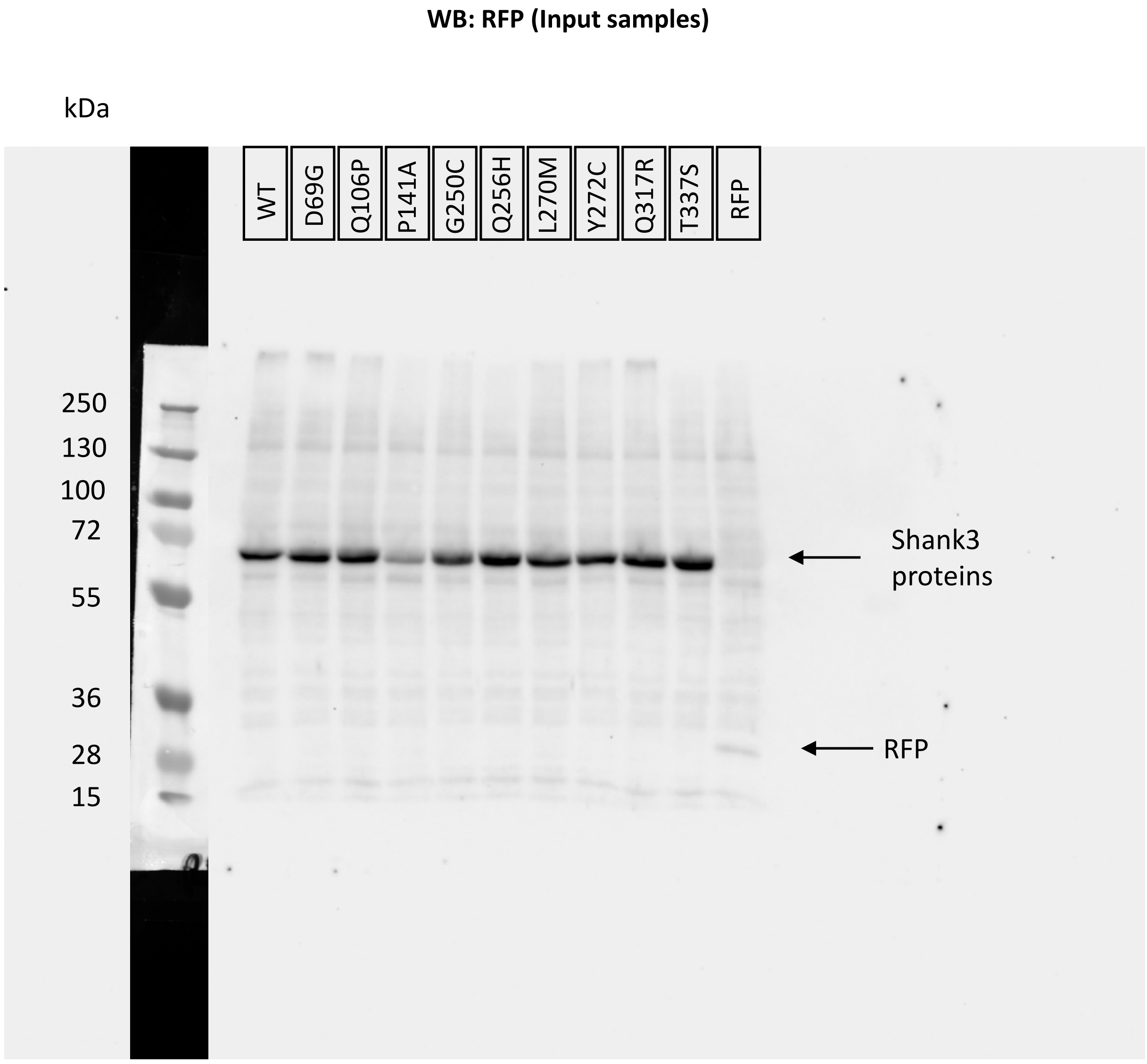
WB: RFP (Input samples)
kDa
WT
D69G
Q106P
P141A
G250C
Q256H
L270M
Y272C
Q317R
T337S
RFP
250
130
100
72
55
36
28
15
Shank3 proteins
RFP

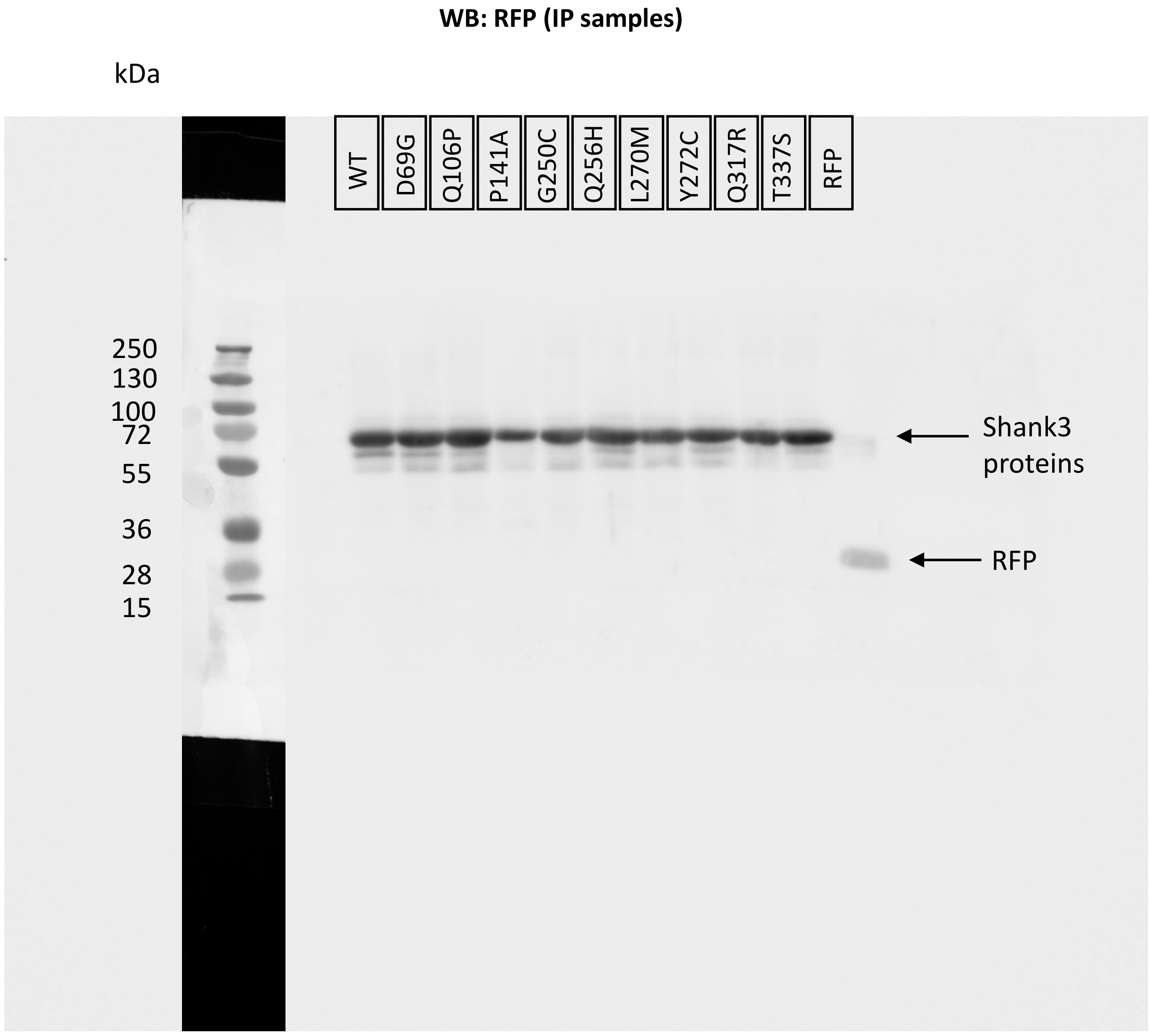
WB: RFP (IP samples)
kDa
WT
D69G
Q106P
P141A
G250C
Q256H
L270M
Y272C
Q317R
T337S
RFP
250
130
100
72
55
36
28
15
Shank3 proteins
RFP

Fig. 4 a.

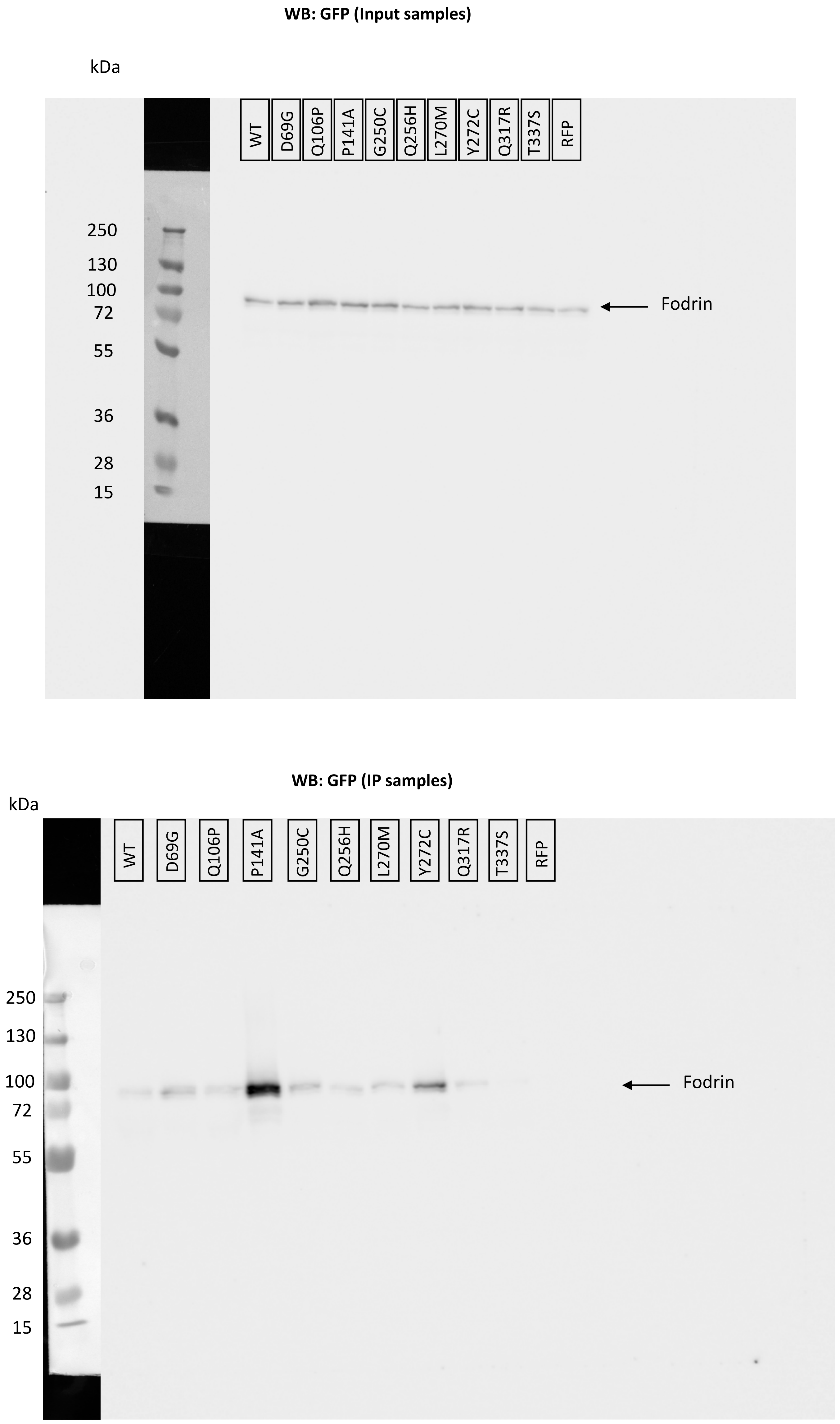

WB: GFP (Input samples)
kDa
WT
D69G
Q106P
P141A
G250C
Q256H
L270M
Y272C
Q317R
T337S
RFP
250
130
100
72
55
36
28
15
Fodrin
WB: GFP (IP samples)
kDa
WT
D69G
Q106P
P141A
G250C
Q256H
L270M
Y272C
Q317R
T337S
RFP
250
130
100
72
55
36
28
15
Fodrin

Fig. 5 a.

WB: RFP (Input samples)

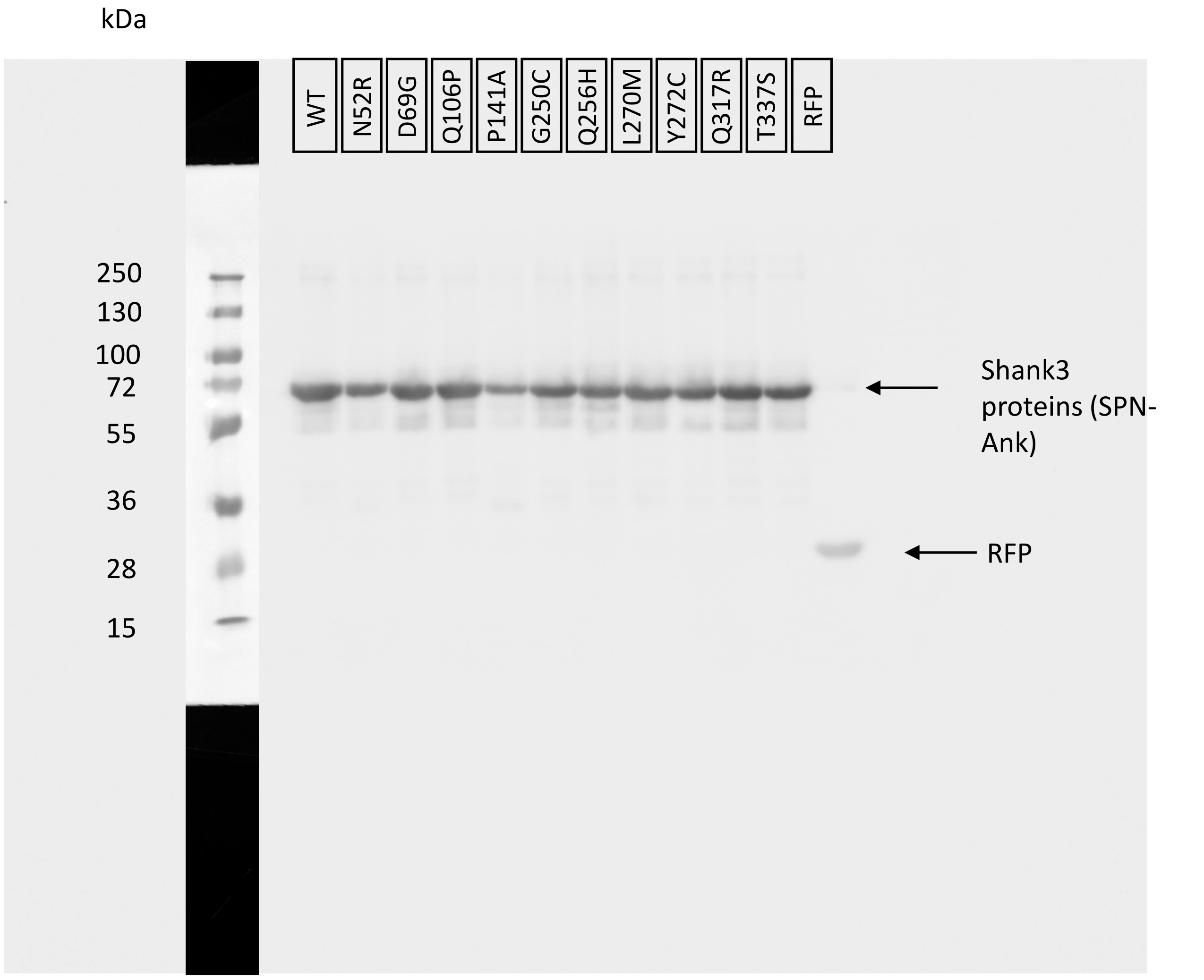

WB: RFP (IP samples)

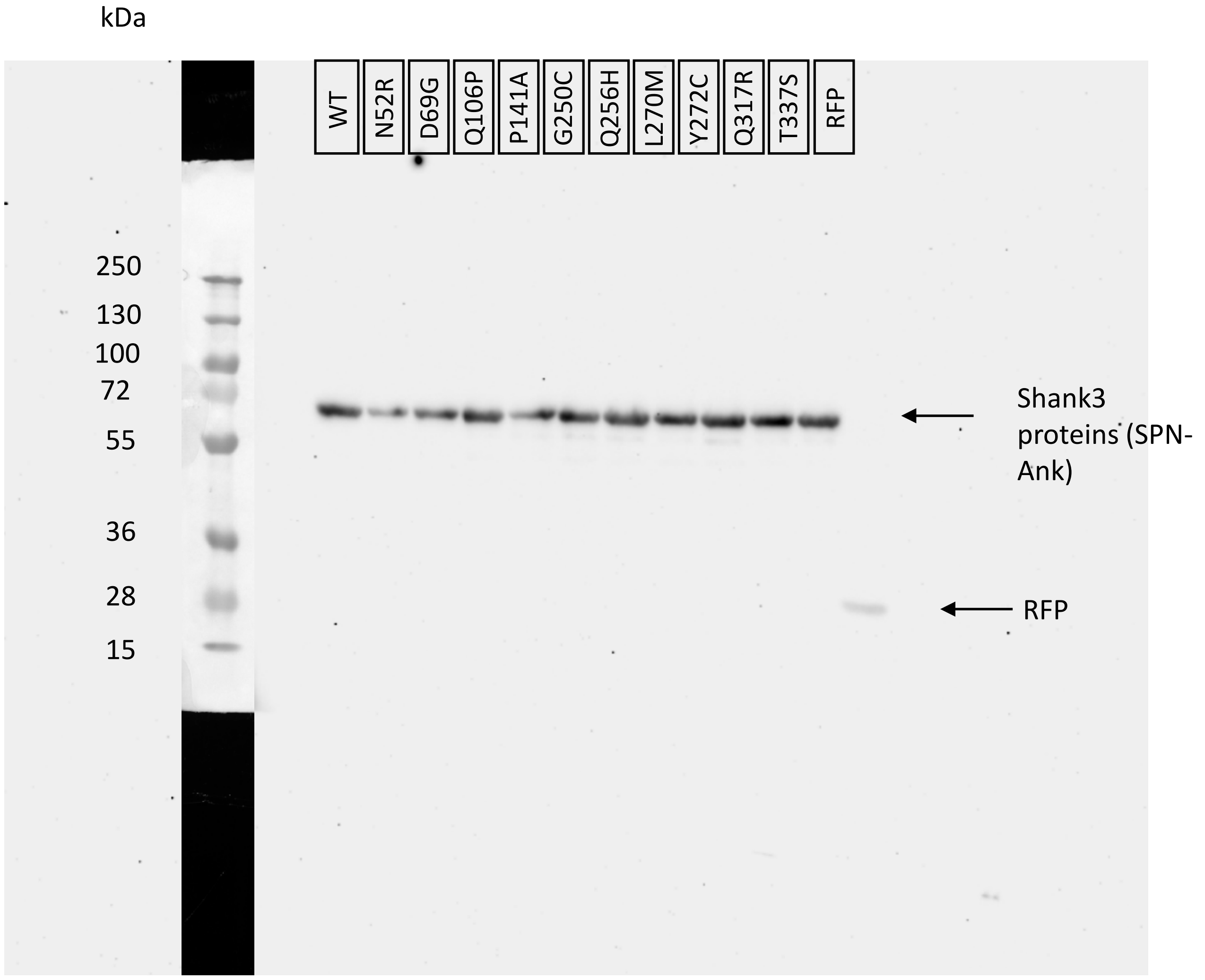

Fig. 5 a.

WB: GFP (Input samples)

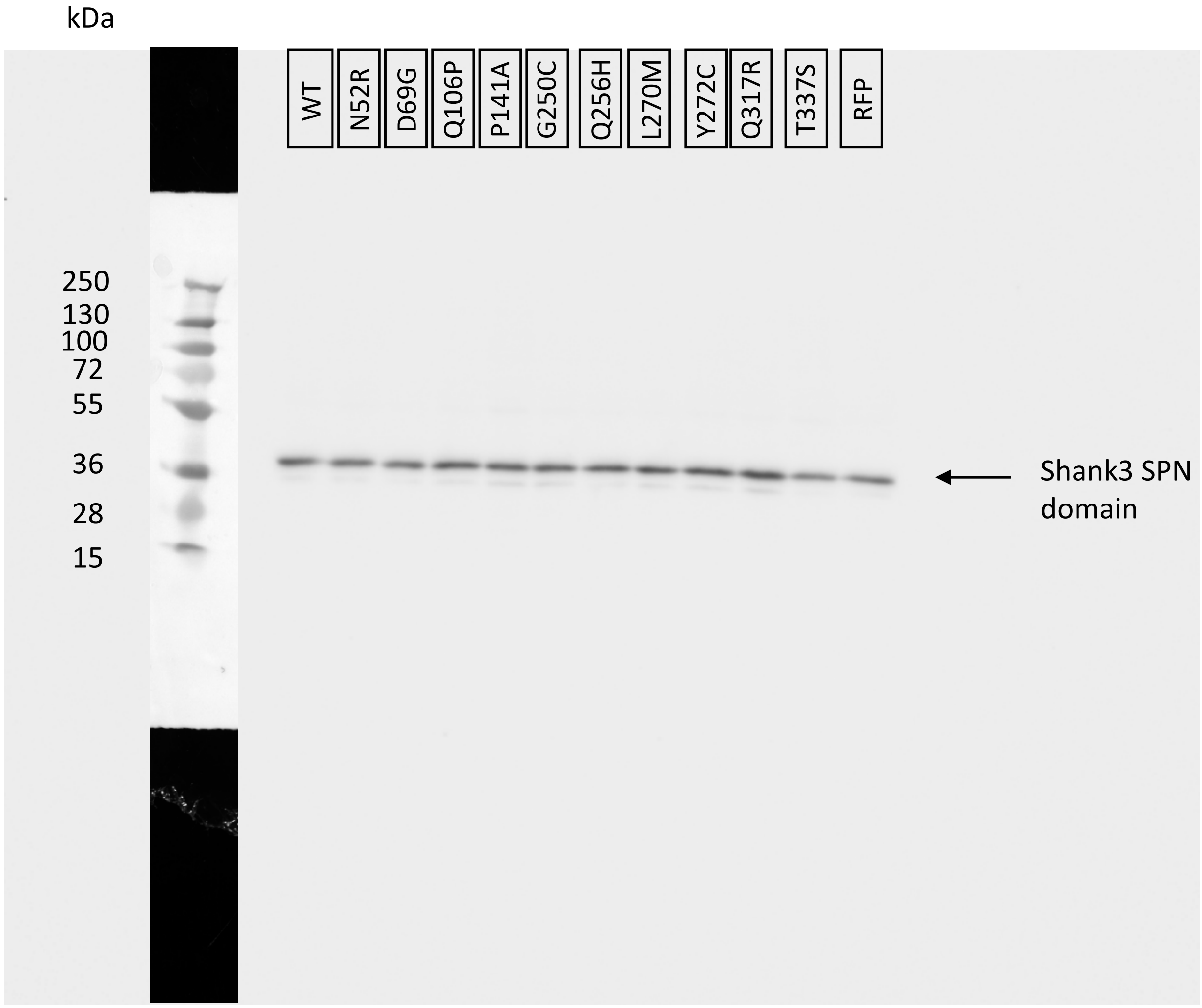

WB: GFP (IP samples)

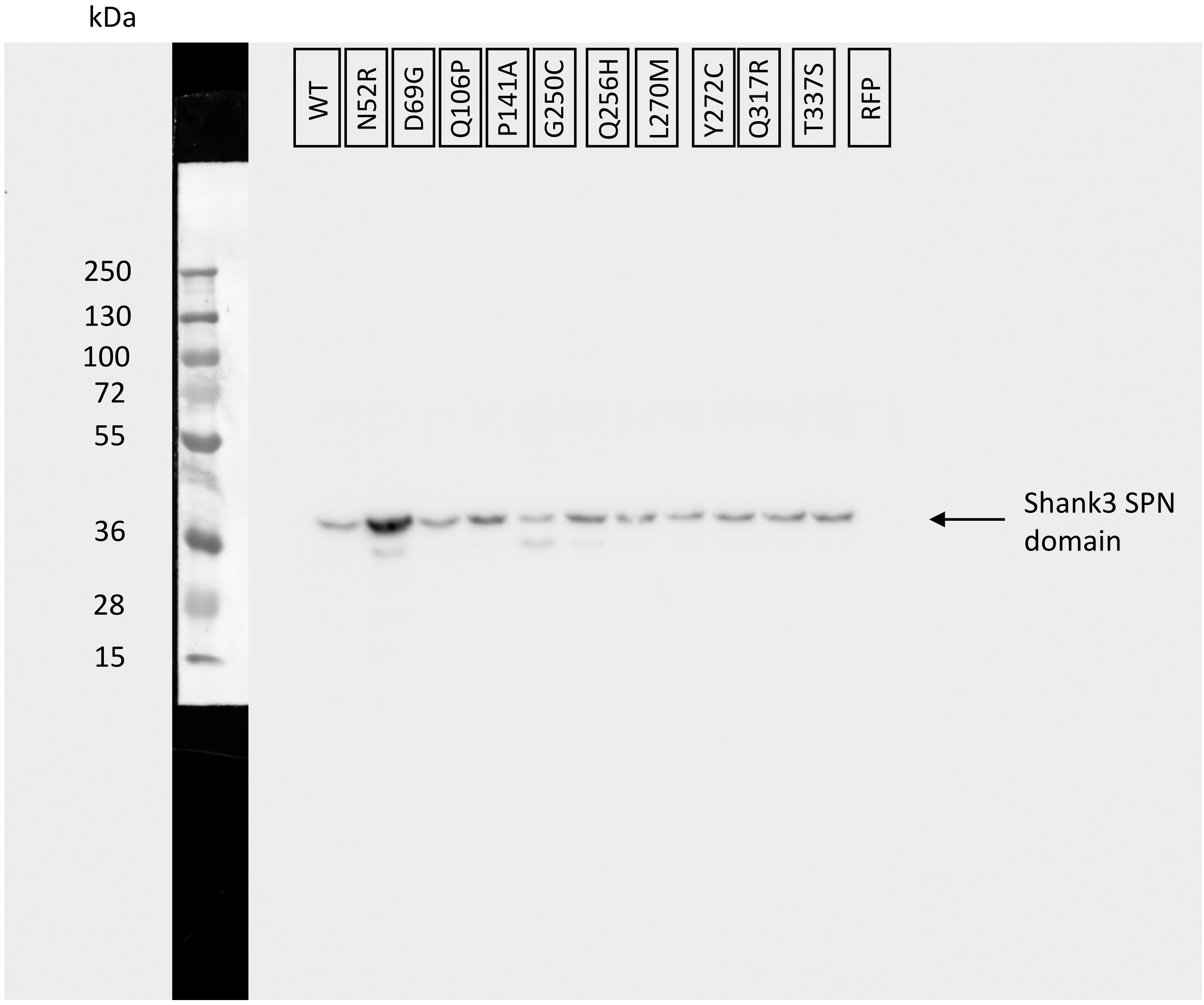

Fig. 5 c.

WB: RFP (Input samples)

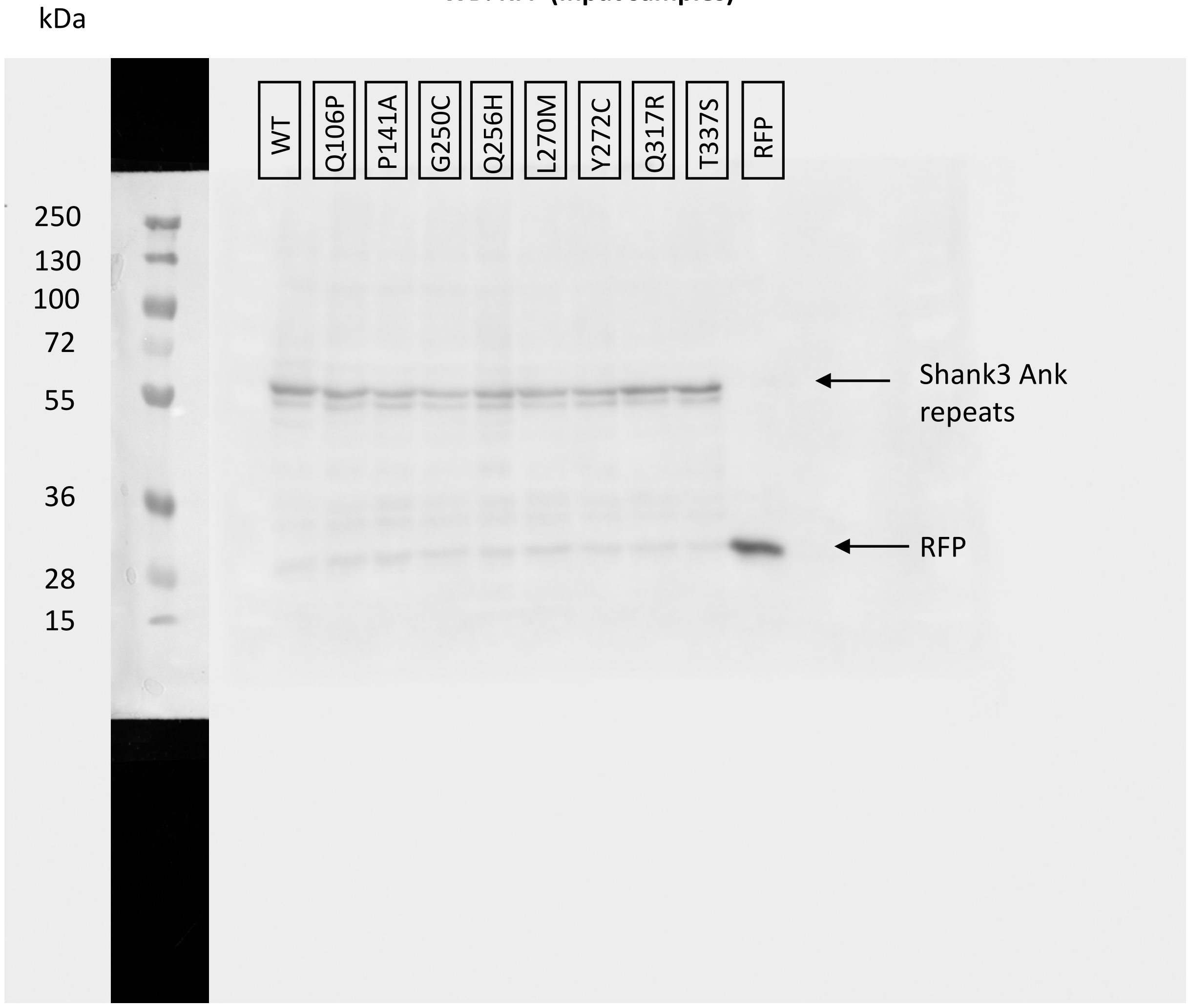

WB: RFP (IP samples)

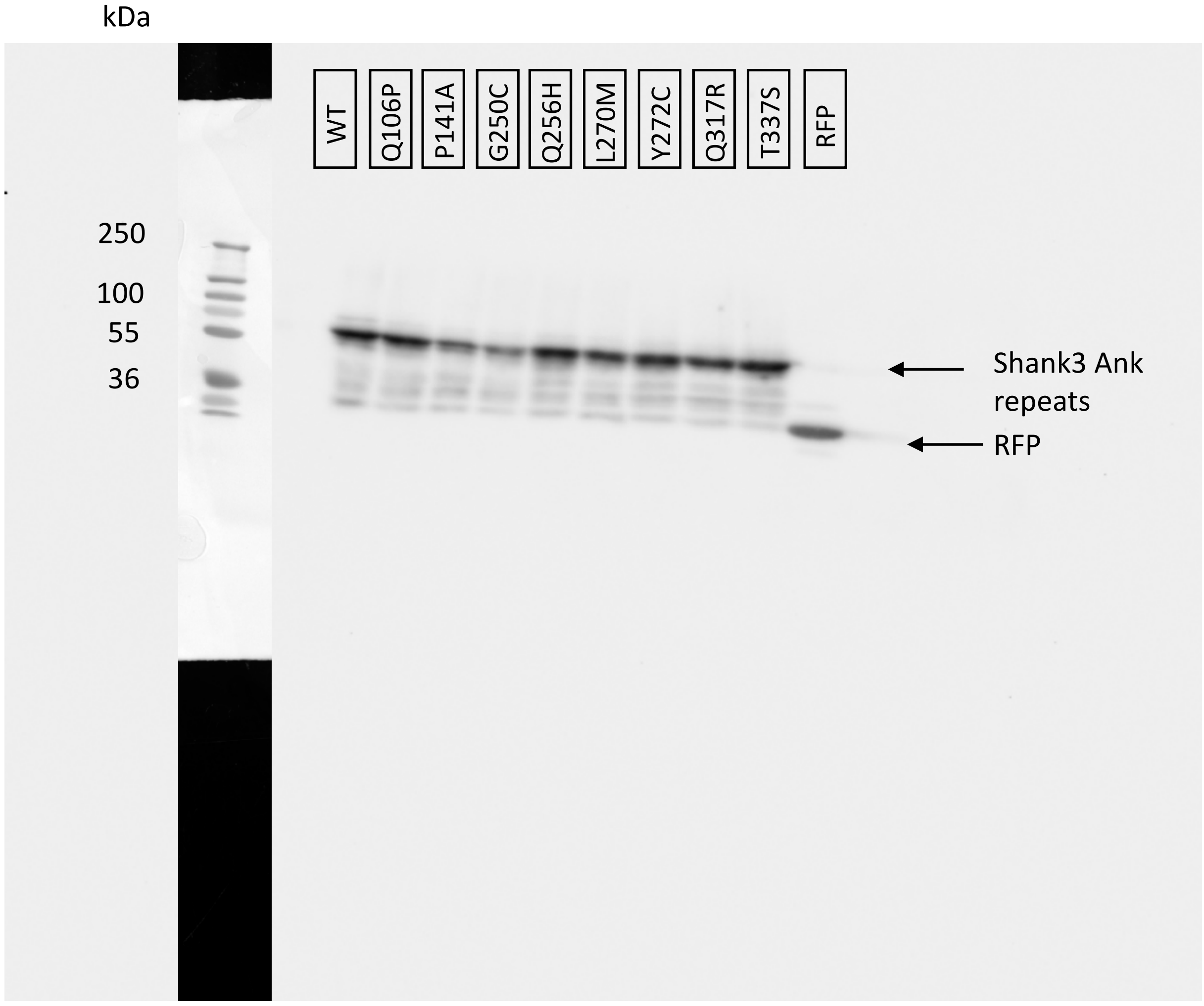

Fig. 5 c.

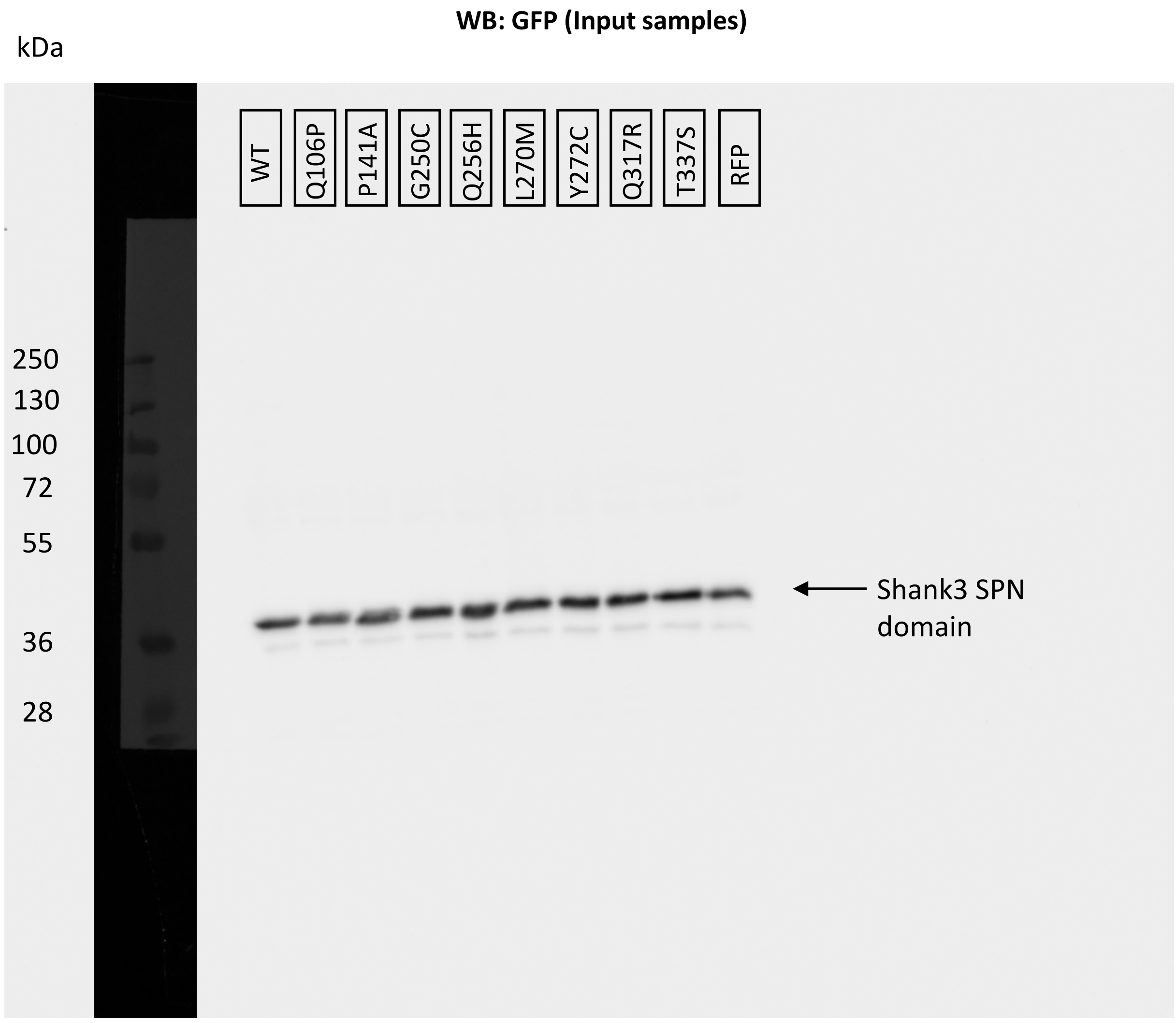
WB: GFP (Input samples)
kDa
WT
Q106P
P141A
G250C
Q256H
L270M
Y272C
Q317R
T337S
RFP
250
130
100
72
55
36
28
Shank3 SPN domain

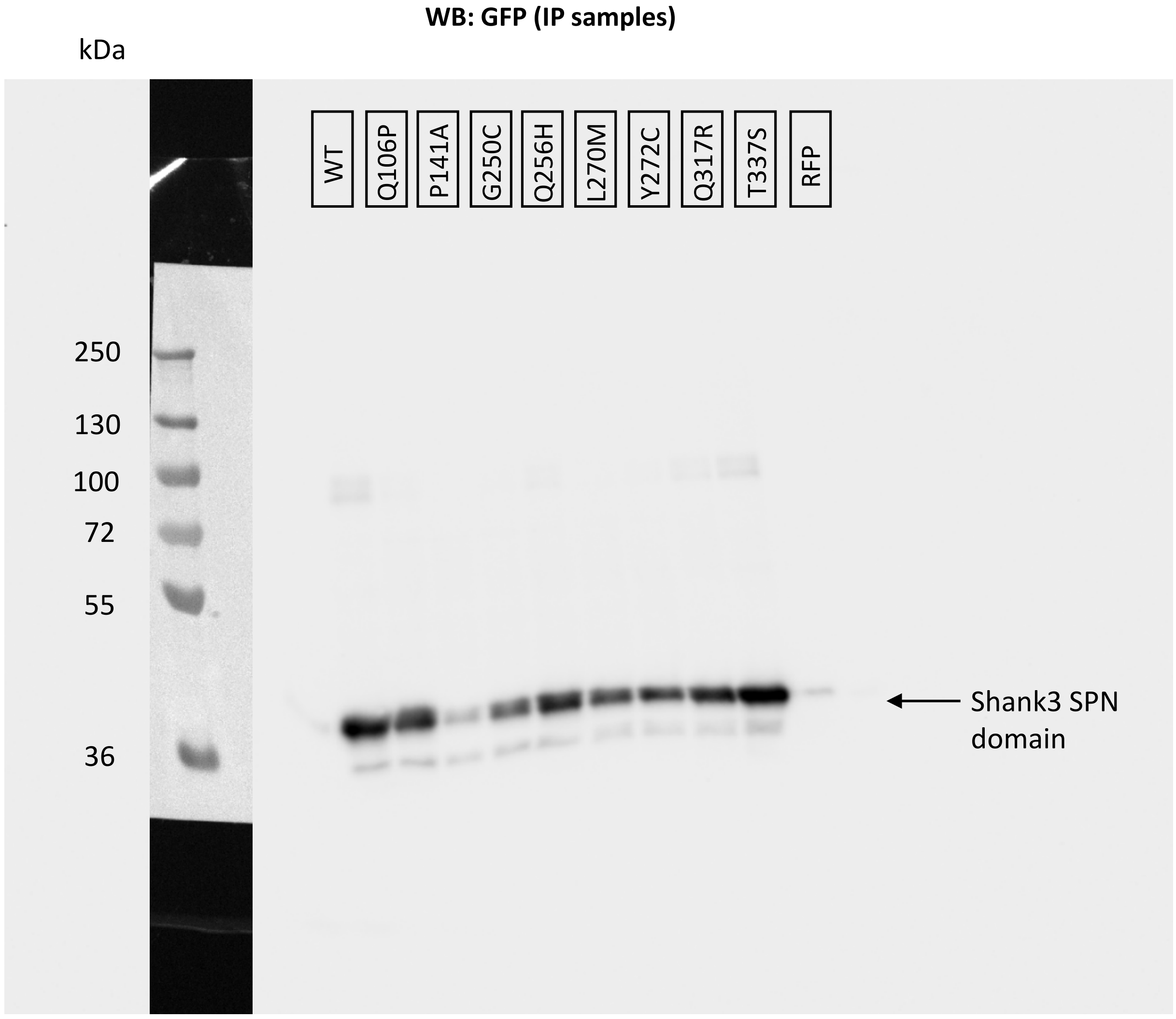
WB: GFP (IP samples)
kDa
WT
Q106P
P141A
G250C
Q256H
L270M
Y272C
Q317R
T337S
RFP
250
130
100
72
55
36
Shank3 SPN domain

Fig. 8 a.

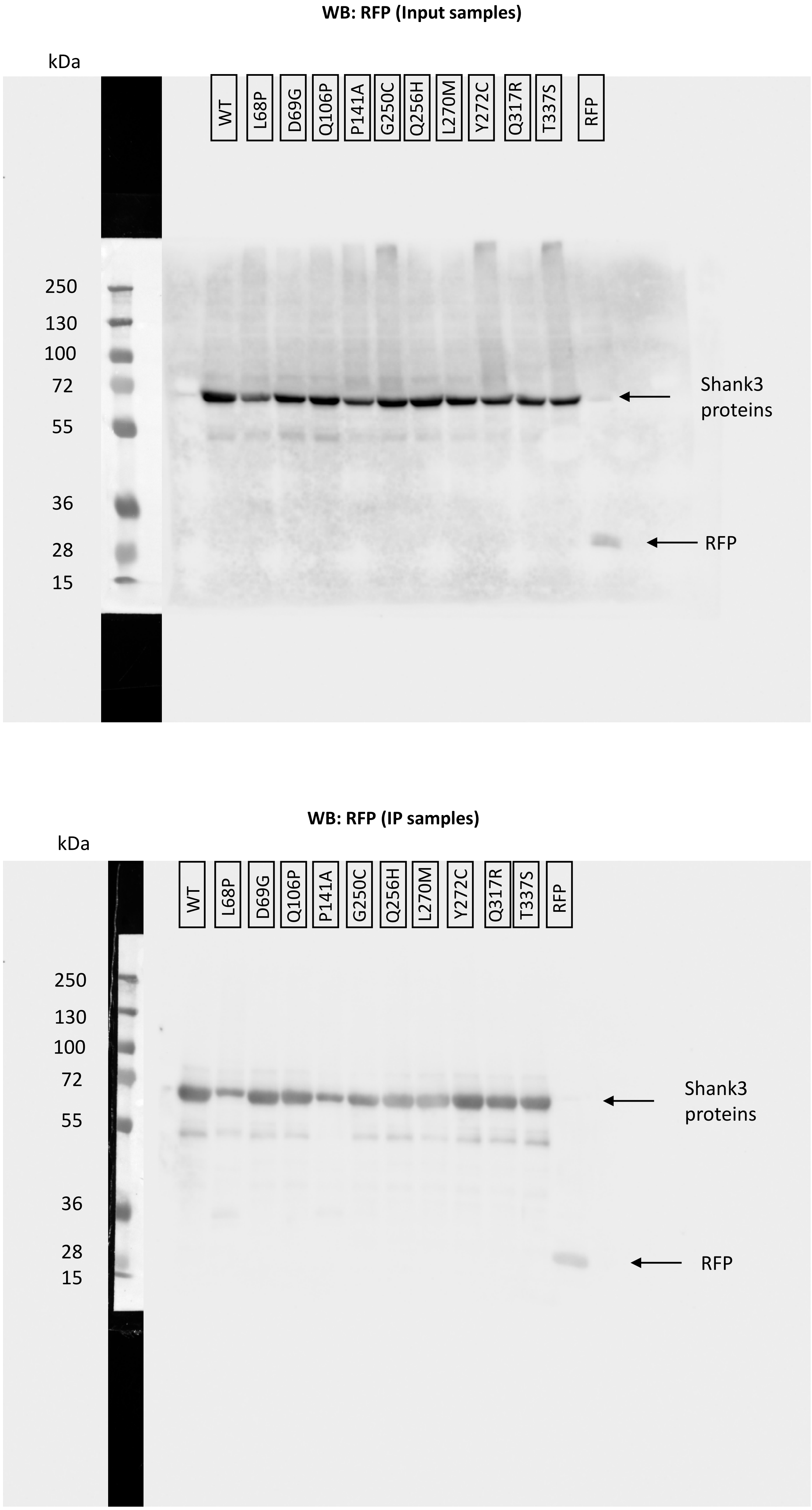
WB: RFP (Input samples)
kDa
WT
L68P
D69G
Q106P
P141A
G250C
Q256H
L270M
Y272C
Q317R
T337S
RFP
250
130
100
72
55
36
28
15
Shank3 proteins
RFP
WB: RFP (IP samples)
kDa
WT
L68P
D69G
Q106P
P141A
G250C
Q256H
L270M
Y272C
Q317R
T337S
RFP
250
130
100
72
55
36
28
15
Shank3 proteins
RFP

Fig. 8 a.

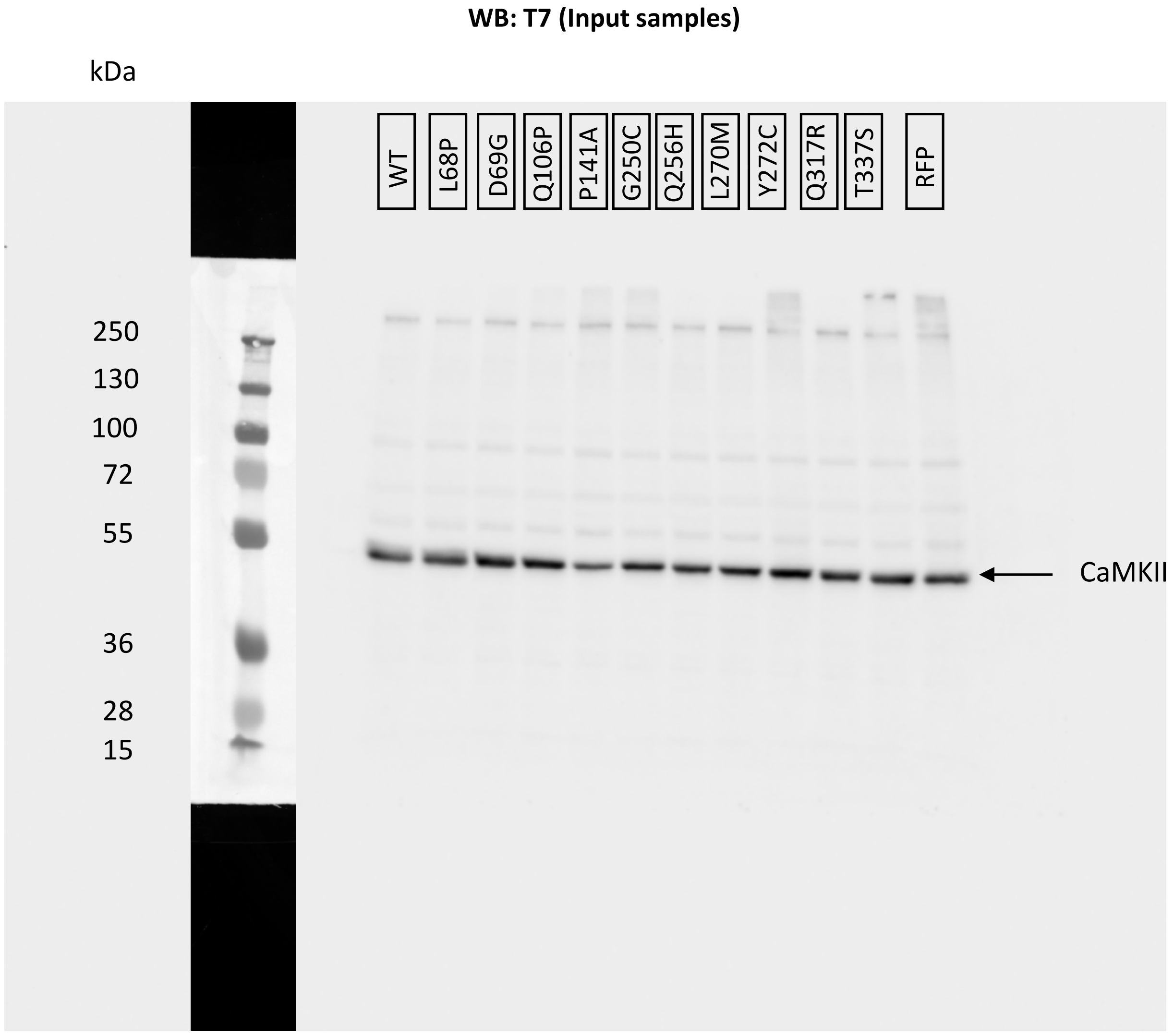
WB: T7 (Input samples)
kDa
WT
L68P
D69G
Q106P
P141A
G250C
Q256H
L270M
Y272C
Q317R
T337S
RFP
250
130
100
72
55
36
28
15
CaMKII

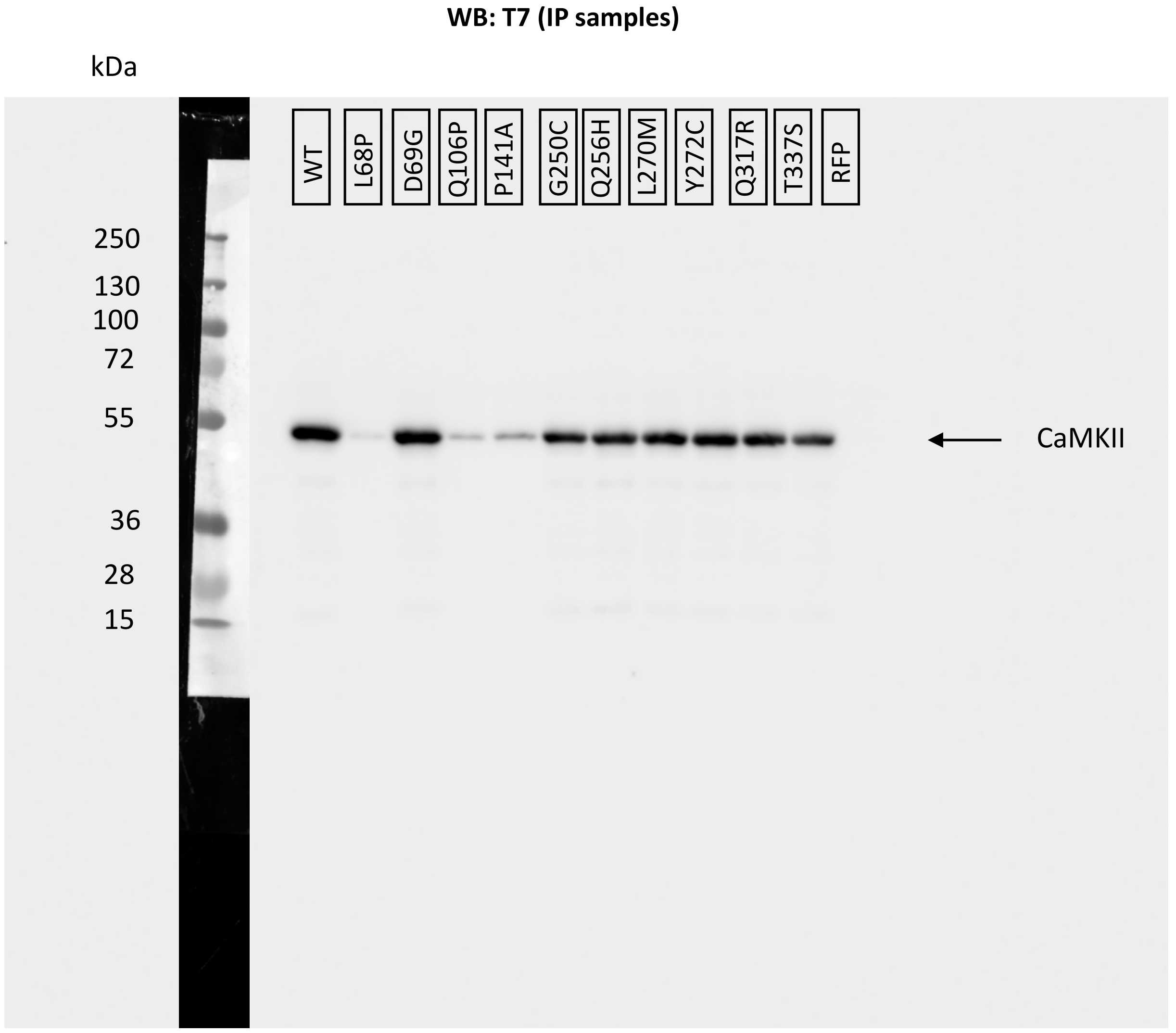
WB: T7 (IP samples)
kDa
WT
L68P
D69G
Q106P
P141A
G250C
Q256H
L270M
Y272C
Q317R
T337S
RFP
250
130
100
72
55
36
28
15
CaMKII

Fig. 8 c.

WB: RFP (Input + IP samples)

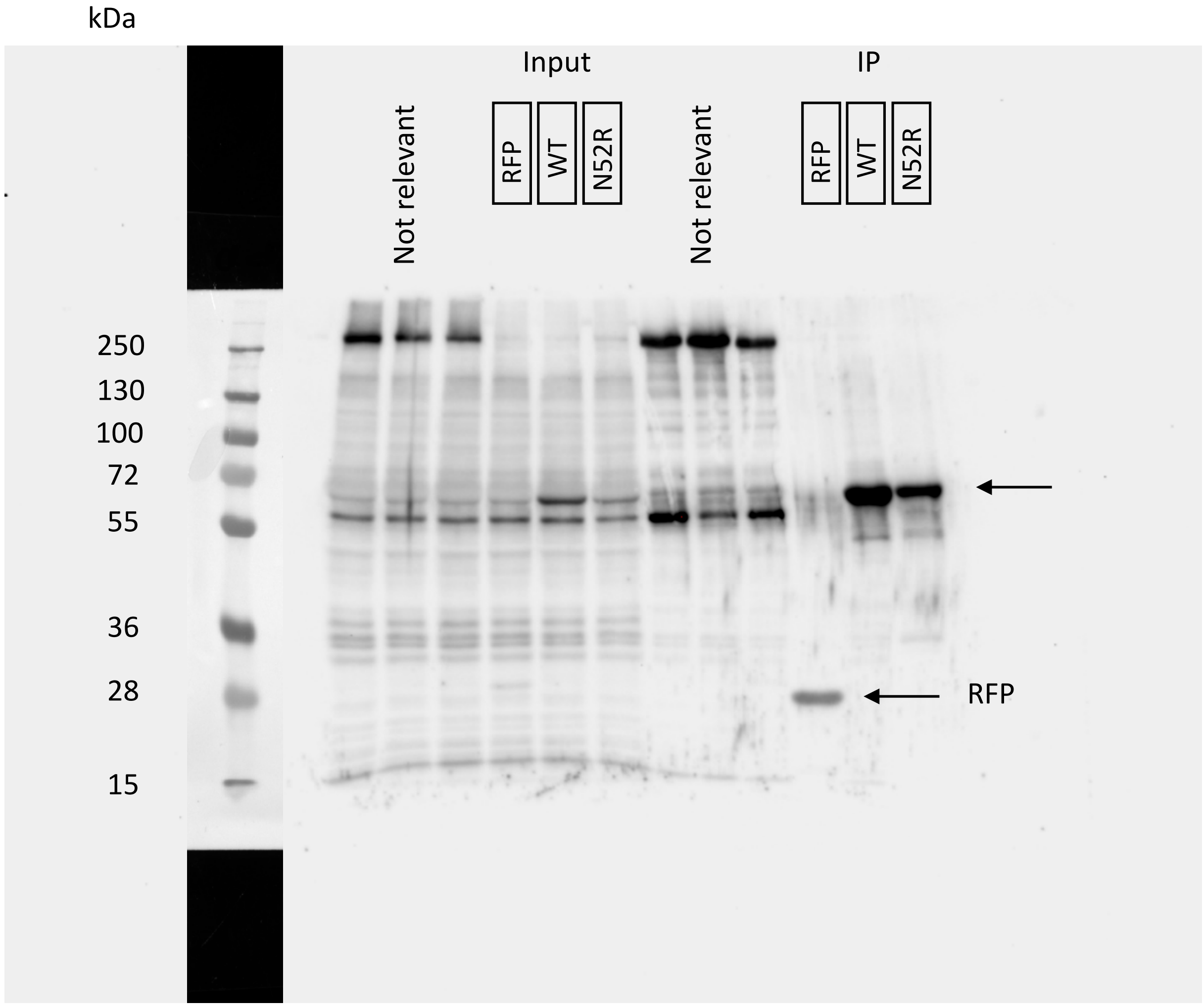

WB: T7 (Input + IP samples)

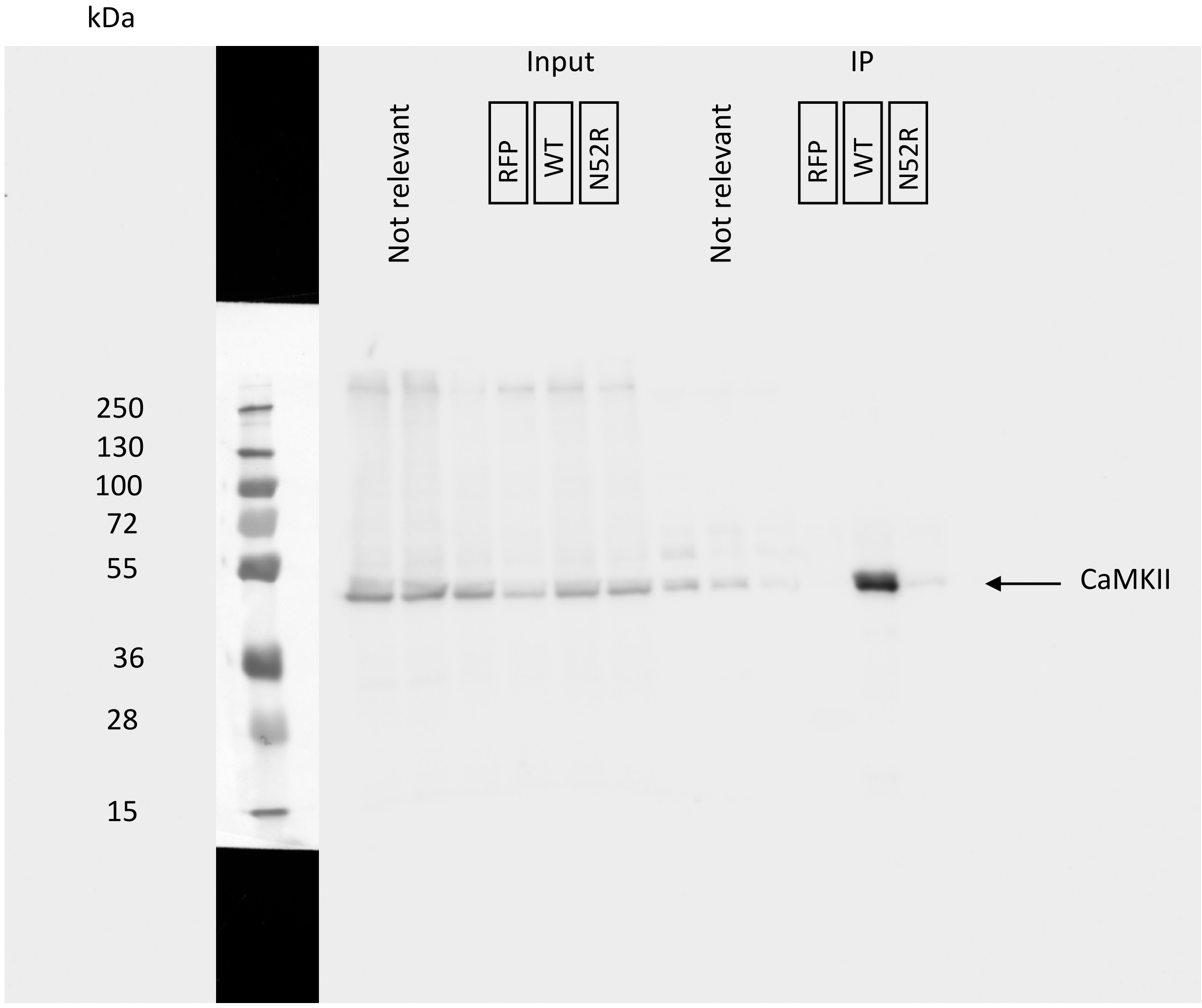

Supplementary Fig. S1 a.

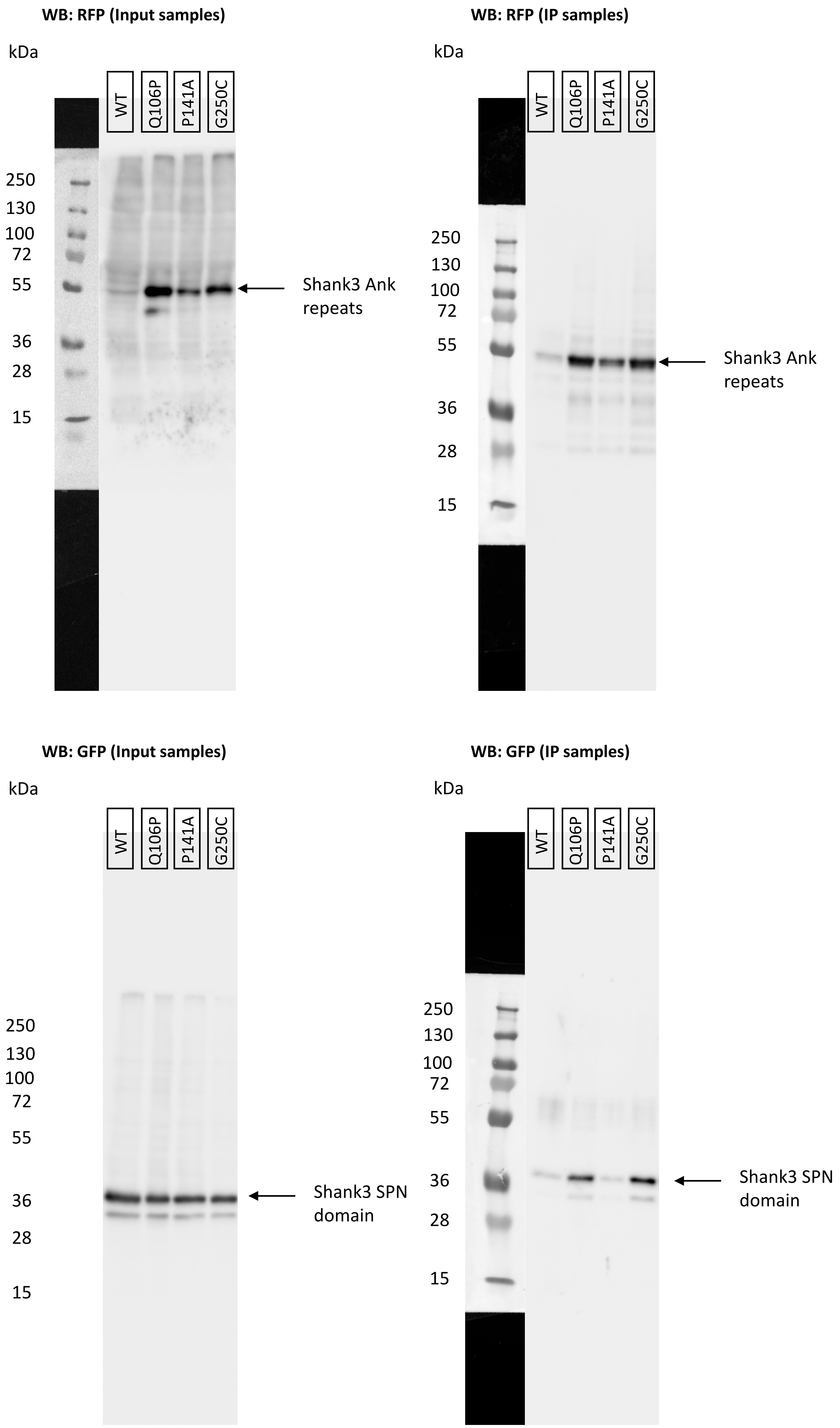

Supplement: Supplementary file 1 — Supplementary Information. [file 41598_2021_4723_MOESM1_ESM.pdf]
